# N-glycan core tri-fucosylation requires Golgi α-mannosidase III activity that impacts nematode growth and behavior

**DOI:** 10.1016/j.jbc.2024.107944

**Published:** 2024-10-29

**Authors:** Jonatan Kendler, Florian Wӧls, Saurabh Thapliyal, Elsa Arcalis, Hanna Gabriel, Sascha Kubitschek, Daniel Malzl, Maria R. Strobl, Dieter Palmberger, Thomas Luber, Carlo Unverzagt, Katharina Paschinger, Dominique A. Glauser, Iain B.H. Wilson, Shi Yan

**Affiliations:** 1 Institut für Parasitologie, Veterinärmedizinische Universität, Wien, Austria; 2 Department für Chemie, Universität für Bodenkultur, Wien, Austria; 3 Department of Biology, University of Fribourg, Fribourg, Switzerland; 4 Department für angewandte Genetik und Zellbiologie, Universität für Bodenkultur, Wien, Austria; 5 Department für Biotechnologie, Universität für Bodenkultur, Wien, Austria; 6 Bioorganic Chemistry, University of Bayreuth, Bayreuth, Germany

**Keywords:** n-glycan, nematode, fucosylation, mannosidase, glycosylation

## Abstract

*N*-glycans with complex core chitobiose modifications are observed in various free-living and parasitic nematodes but are absent in mammals. Using *Caenorhabditis elegans* as a model, we demonstrated that the core *N*-acetylglucosamine (GlcNAc) residues are modified by three fucosyltransferases (FUTs), namely FUT-1, FUT-6, and FUT-8. Interestingly, FUT-6 can only fucosylate *N*-glycans lacking the α1,6-mannose upper arm, indicating that a specific α-mannosidase is required to generate substrates for subsequent FUT-6 activity. By analyzing the *N*-glycomes of *aman-3* KOs using offline HPLC-MALDI-TOF MS/MS, we observed that the absence of *aman-3* abolishes α1,3-fucosylation of the distal GlcNAc of *N*-glycans, which suggests that AMAN-3 is the relevant mannosidase on whose action FUT-6 depends. Enzymatic characterization of recombinant AMAN-3 and confocal microscopy studies using a knock-in strain (*aman-3::eGFP*) demonstrated a Golgi localization. In contrast to the classical Golgi α-mannosidase II (AMAN-2), AMAN-3 displayed a cobalt-dependent α1,6-mannosidase activity toward *N*-glycans. Using AMAN-3 and other *C. elegans* glycoenzymes, we were able to mimic nematode *N*-glycan biosynthesis *in vitro* by remodeling a fluorescein conjugated-glycan and generate a tri-fucosylated structure. In addition, using a high-content computer-assisted *C. elegans* analysis platform, we observed that *aman-3* deficient worms display significant developmental delays, morphological, and behavioral alterations in comparison to the WT. Our data demonstrated that AMAN-3 is a Golgi α-mannosidase required for core fucosylation of the distal GlcNAc of *N*-glycans. This enzyme is essential for the formation of the unusual tri-fucosylated chitobiose modifications in nematodes, which may play important roles in nematode development and behavior.

Protein glycosylation is a ubiquitous and evolutionarily conserved posttranslational modification observed across diverse species. It exerts pleiotropic effects on many biological phenomena under both physiological and pathophysiological conditions. As an important modulator of signaling, cell adhesion, and cell-cell interactions, protein glycosylation profoundly influences embryogenesis, tissue homeostasis, as well as cancer progression (1, 2, 3). The nervous system also relies on precise glycosylation for proper neuronal development and function (4). Moreover, glycoconjugates of pathogens are the major determinants that trigger immune responses, as they often possess ligands for innate immune recognition and promote production of anti-glycan antibodies (5, 6).

*Caenorhabditis elegans* has been used as a good model to study the glycosylation patterns conserved among parasitic species. In contrast to mammals, it has been shown that *C. elegans* can express complicated core modified N-glycan structures and a portion of these structural elements (glyco-epitopes) can also be found in parasitic nematodes (7). Some nematode glycoepitopes are obviously immunogenic in mammals, for instance the anti-horseradish peroxidase epitope (core α1,3-fucose) is recognized by immunoglobulins (Igs) IgE and IgG antibodies of the host (8, 9).

Correct glycosylation of an antigen is considered, in addition to the protein backbone, an important factor for the antigenicity of parasite proteins. Therefore, identifying the correct glycoepitopes and glycan structures on the native glycoproteins and characterizing the glyco-enzymes involved in the biosynthesis are two major challenges for glycobiologists.

In our previous work, we have proven the function of the three fucosyltransferases (FUTs), which are involved in the biosynthesis of the highly fucosylated N-glycan core in *C. elegans*: FUT-1 and FUT-8, respectively, direct the α1,3- and α1,6-fucosylation of the proximal *N*-acetylglucosamine (GlcNAc) residue (10, 11), whereas FUT-6 is responsible for α1,3-fucosylation of the distal GlcNAc residue (summarized in Fig. 1) (12). Homologs of *C. elegans* FUT-8 have been well-studied in both vertebrates and invertebrates (11, 13, 14); enzymes with the same function as FUT-1 are characterized from plants and invertebrates (15), whereas FUT-6 (12) is a unique core FUT occurring in some nematodes. It is noteworthy that FUT-6 displayed a strong bias against the presence of the α1,6-mannose residue (known as the ”upper-arm”) in contrast to the α1,3-mannose residue (known as the “lower-arm”) of N-glycan structures as judged by the results of *in vitro* enzymatic assays (12); in other words, FUT-6 only fucosylated the structures lacking the α1,6-linked mannose. Analyzing the glycan structures from WT and *fut-6* deficient mutants as well as a double hexosaminidase mutant (*hex-2;hex-3*) yielded further evidence for this property of FUT-6 (16).

**Figure 1:**
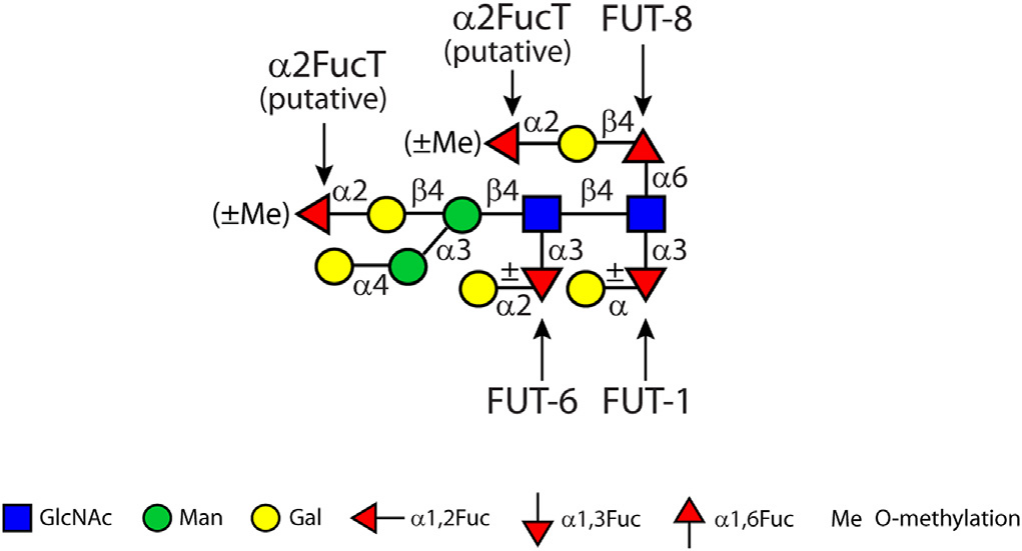
**An illustration of fucose-rich glycans of the WT *Caenorhabditis elegans* using the Symbol Nomenclature for Glycans (SNFG)** (49)**.** Known fucosyltransferases (FUTs) as well as putative α1,2-fucosyltransferases (α2FucTs) responsible for fucosylation of the N-glycan core region with the specific glycosidic linkage are indicated with *arrows*.

In addition to the core difucosylation of the proximal GlcNAc residue, distal GlcNAc fucosylation seems to be conserved in a number of nematode species, such as *Pristionchus pacificus*, *Ascaris suum*, *Oesophagostomum dentatum*, and *Haemonchus contortus* (16, 17, 18, 19), probably due to the activity of FUT-6 orthologues, but are absent from filarial worms or *Trichuris suis* (9, 20). In the case of *Haemonchus*, a predicted glycosyltransferase (Genbank accession number: CDJ84058.1) possesses the highest homology to *C. elegans* FUT-6 in comparison to other FUT homologs. Consistent with the *in vitro* properties of FUT-6 (see above), detailed N-glycan structural analyses of these nematodes suggested the absence of the α1,6-mannose residue on the glycans which carry either solely an α1,3-linked fucose or a galactosylated fucose disaccharide unit on the distal GlcNAc. Presumably, the “upper arm” generates a steric hindrance which restricts the access of FUT-6 to the 3-OH position of the distal GlcNAc. These observations led to the assumption that a Golgi α1,6-mannosidase activity must be essential for a proper biosynthesis of such structures prior to further processing by FUT-6 homologs.

There are three glycosyl hydrolase GH38 homologs in *C. elegans*: one lysosomal (AMAN-1), one classical Golgi mannosidase II (AMAN-2), and a hypothetical mannosidase (AMAN-3). In insects, enzymes with similarities to the latter (mannosidase III or ManIIb) have been characterized and shown to have Co(II)-dependent activities toward N-glycans (21, 22). In contrast to AMAN-2 that unequivocally impacts *C. elegans* N-glycan biosynthesis, it remained unclear if AMAN-3 can process N-glycoproteins despite its activity toward an artificial substrate in the presence of Cobalt(II) chloride (23). By BLAST search using the extracellular domain of *C. elegans* AMAN-3, α-mannosidase III homologs can also be found in *H. contortus* (CDJ83252.1) and *A. suum* (ERG79326.1) with 39% and 44% identity, respectively. Therefore, it is highly possible that in addition to FUT-1 and FUT-8 homologs, these nematodes also express α-mannosidase III and FUT-6 homologs, which may sequentially act on N-glycans to create the highly fucosylated core structures found in these species.

In this study, we systematically investigated the enzymatic properties of the *C. elegans* AMAN-3 and proved its involvement in N-glycan biosynthesis. AMAN-3 deficiency not only impacts the core fucosylation patterns of nematode N-glycoproteins, but also alters animal development and results in behavioral changes.

## Results

### α1,6-specific mannosidase activity is lacking in aman-3 mutant worm lysates

**Figure 2:**
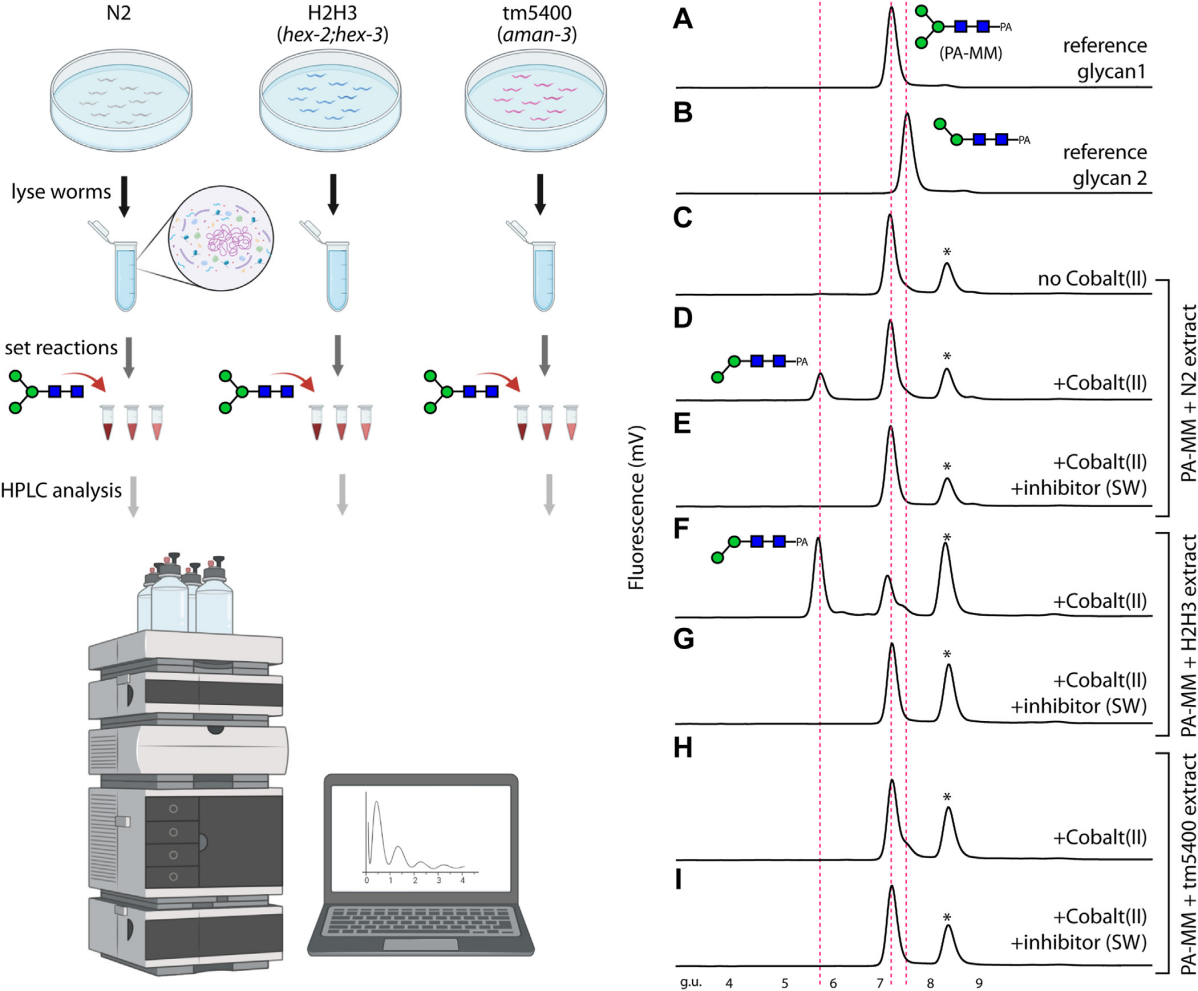
**Detection of AMAN-3 activity in the crude extracts of different *Caenorhabditis elegans* strains.** Worms were lysed to release native enzymes and clear supernatants were used to digest a pyridylamino-labeled N-glycan under various conditions; reaction mixtures were analyzed on reversed phase HPLC (*left panel*, created with BioRender.com). A selected N-glycan (*A*, PA-MM, 7.2 g.u.) was incubated with worm supernatants prepared from either the WT (N2; *C–E*), *hex-2;hex-3* double KO (H2H3; *F* and *G*) or an *aman-3* single KO (tm5400; *H* and *I*) with/without the presence of Cobalt (II) or Cobalt (II) plus swainsonine (SW). (*B*) shows the elution of a less improtant reference compound that was used to aid structural analysis. The presence of an early eluting product, missing the α1,6-linked mannose, on HPLC chromatograms (*D* and *F*) indicated the α-mannosidase III activities. This cobalt (II)-dependent demannosylation was only observed when N2 or H2H3 extract was used but was absent when the tm5400 extract was used (*H*). Peaks marked with an *asterisk* contain nonglycan contaminants; g.u. is an abbreviation of glucose units. The peaks were all analyzed by MALDI-TOF MS and the major Co(II)-dependent product at 5.7 g.u. shown to have a glycan of *m/z* 827 [M+H]^+^ as compared to the substrate of *m/z* 989.

In a previous study, we showed that the unpurified recombinant product of *C. elegans* F48C1.1 or *aman-3* gene could degrade *p*-nitrophenyl-α-mannoside and potentially cause degradation of pyridylaminated Man_5_GlcNAc_2_ (23). In order to confirm the Co(II)-dependent mannosidase activity of native AMAN-3, verify its sensitivity to the swainsonine inhibitor and better delineate its N-glycan substrate specificity, we conducted additional analyses to assess how a selected N-glycan (Man_3_GlcNAc_2_, 2-aminopyridine labelled MM structure [PA-MM] in Fig. 2) is processed by lysates from WT worms (N2), *aman-3* loss-of-function mutants (tm5400), and *hex-2;hex-3* mutants with deletions in two Golgi hexosaminidase genes (16). Noteworthily, the *hex-2;hex-3* mutant is known to possess high amount of N-glycan structures missing the α1,6-mannose upper-arm, presumably due to a high α1,6-specific mannosidase activity in this mutant. At approximately neutral pH, a removal of one mannose from PA-MM was observed with N2 and *hex-2;hex-3* lysates (Fig. 2, *D* and *F*), but not for the *aman-3* lysate (Fig. 2, *H* and *I*); this is evidenced by the appearance of a new HPLC peak at 5.7 g.u. and confirmed by MALDI-TOF-MS (loss of 162 Da). Based on the observed mass change and the forward shift in elution time, the Man_2_GlcNAc_2_ product was identified as Manα1,3Manβ1,4GlcNAcβ1,4GlcNAc-PA (17, 24). We conclude that the *aman-3* mutant lacked an Co(II)-dependent, swainsonine-inhibitable α1,6-specific mannosidase activity.

### N-glycomes of aman-3 deficient mutants

**Figure 3:**
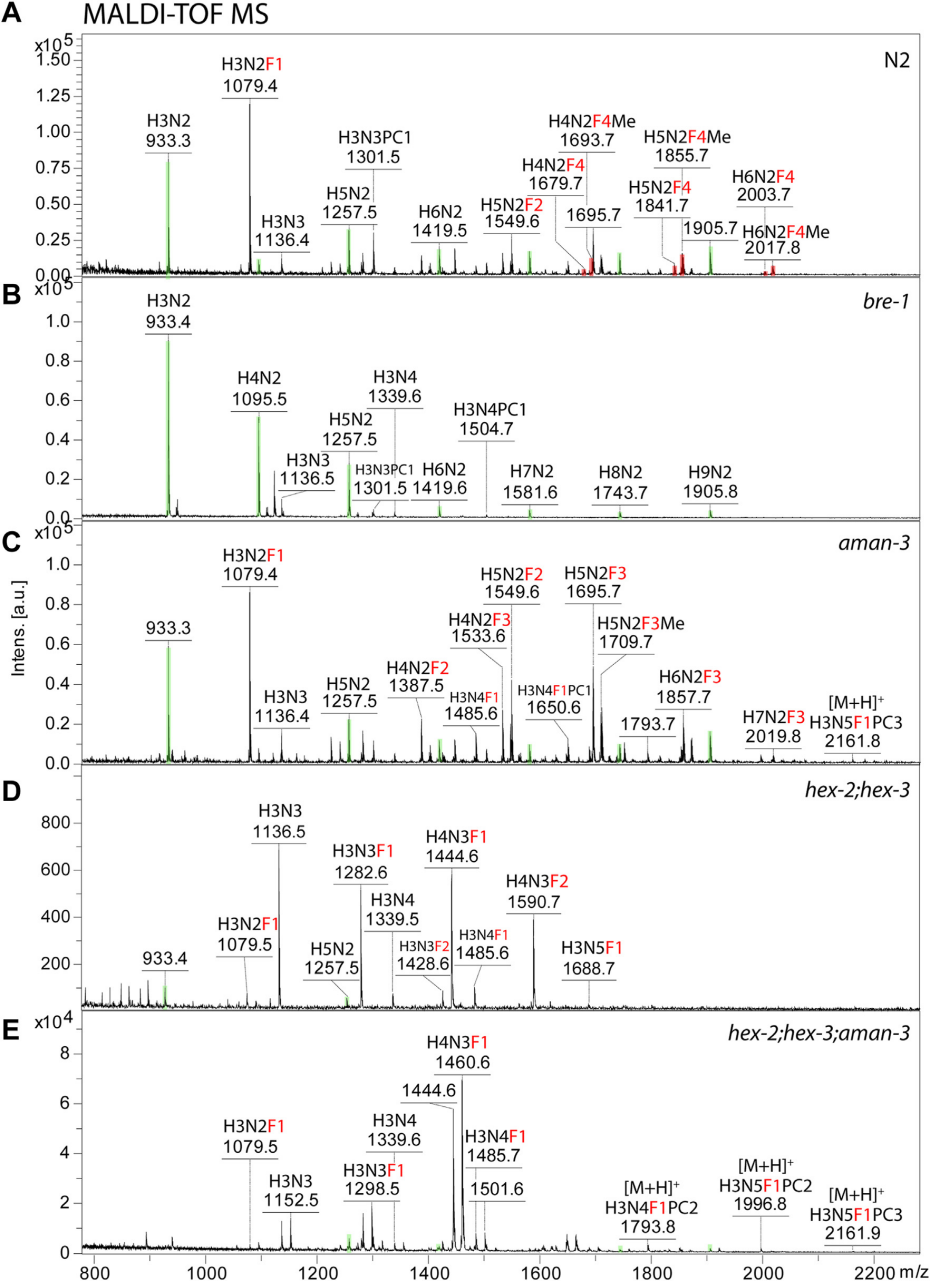
**MALDI-TOF MS spectra of native N-glycans from the WT and KO strains.** N-glycans released by PNGase A were subject to solid phase extraction and MS analysis. Most of the glycans present on the spectra are in [M+Na]^+^ and [M+K]^+^ forms except for PC-modified glycans detected partly in [M+H]^+^ forms. Peaks are annotated with *m/z* values and glycan compositions (H, hexose; N, *N*-acetyl-hexosamine; F, fucose, highlighted in *red*; Me, methyl group; PC, phosphorylcholine). Peaks indicating pauci- and oligo-mannosidic glycans are highlighted in *green*. In comparison to the N2 WT that possesses tetrafucosylated N-glycans (*A*), underfucosylation with maximal three fucoses is observed in *aman-3* single mutant (*C*). In comparison to the H2H3 double mutant (*D*) (16), *hex-2;hex-3;aman-3* triple knockout (*E*) possesses three major compositions (H_3-4_N_3_F_0-1_) and the difucosylated glycan H_4_N_3_F_2_ (*m/z* 1590) is absent on the spectrum (the fourth and fifth panels). *Bre-1* (28) is a fucose-free mutant deficient in GDP-mannose 4,6-dehydratase (*B*). MS, mass spectrometry.

Many N-glycans in WT *C. elegans* lack the α1,6-mannose residue as well as a “lower arm” β1,2-GlcNAc residue on the α1,3-mannose (25, 26, 27), whereas the *hex-2;hex-3* double mutant has high amounts of glycans lacking the α1,6-mannose, but presenting the nonreducing GlcNAc residue (16); on the other hand, the *bre-1* mutant lacks fucosylated glycans due to a mutation in the GDP-Man dehydratase gene (28). Therefore, considering the hypothesis that AMAN-3 was an α1,6-specific mannosidase that may impact fucosylation, we compared WT (N2), *hex-2;hex-3* and *bre-1* strains with an *aman-3* mutant (tm5400) and a *hex-2;hex-3;aman-3* triple mutant (cop1842). Distinct and major shifts in the PNGase A-released N-glycome were observed for all mutants examined and up to three fucose residues were detected in the overall MALDI-TOF MS profile for the *aman-3* mutant but only one fucose in the *hex-2;hex-3;aman-3* triple mutant (Fig. 3), whereby the major N-glycans in these two strains were respectively, Hex_5_HexNAc_2_Fuc_2-3_ and Hex_4_HexNAc_3_Fuc_1_. In line with a previous study (28), no fucosylated N-glycan was detected in the N-glycome of *bre-1*, which suggested the complete absence of core and antennal fucose in this mutant (Fig. 3*B*).

**Figure 4:**
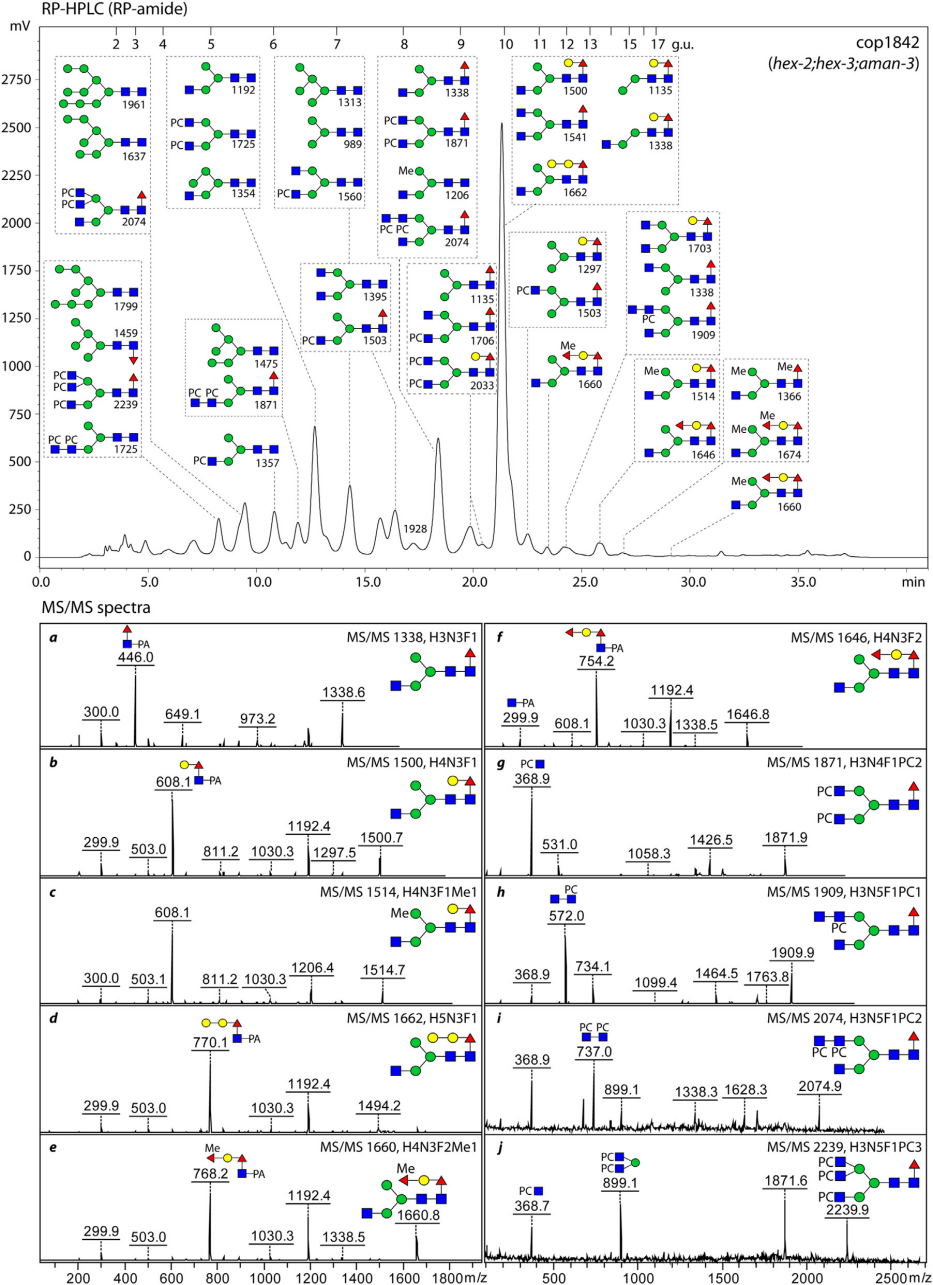
**RP-HPLC chromatogram and MS/MS spectra of PNGase A-released N-glycans from the *hex-2;hex-3;aman-3* triple KO (cop1842).** PA-labeled glycans were fractionated on an RP-amide column and analyzed by MALDI TOF MS/MS. Based on their elution patterns (glucose units) and MS data, predominant glycan structures detected in major HPLC peaks and key B/Y fragment ions are annotated with SNFG format and *m/z* values ([M+H]^+^). Structural assignments are on the basis of MS/MS as well as comparisons to other studies using the same HPLC column (19, 27). MS/MS, tandem mass spectrometry; SNFG, Symbol Nomenclature for Glycans; Me, methyl; PC, phosphorylcholine.

To investigate the N-glycomic changes more exactly, HPLC was performed on a fused core RP-amide column and fractions were individually subject to MALDI-TOF tandem mass spectrometry (MS/MS) (Fig. 4). The major glycan in the triple mutant (Hex_4_HexNAc_3_Fuc_1_; *m/z* 1500 as a pyridylaminated glycan) possessed a “GalFuc” motif, due to β1,4-galactosylation of the core α1,6-fucose by GALT-1 (29), resulting in a strong Y1 fragment at *m/z* 608 (Fig. 4*B*). The traces of glycans with two fucose residues were due to α1,2-fucosylation of the GalFuc (Y1 fragment at *m/z* 754), an epitope previously found in some FUT mutants (30). The high degree of substitution of the α1,3-mannose by the β1,2-GlcNAc residue in the triple mutant is probably the reason for the lack of methylation or α-galactosylation of the α1,3-mannose as well as the absence of bisecting galactose or reducing terminal core α1,3-fucose found in WT N-glycomes (25, 31); in contrast, methylation of the α1,6-mannose was observed (see glycans of, *e.g.*, *m/z* 1206 and 1514; Fig. 4). As complex glycosylation was not blocked, but perhaps even preferred, a range of phosphorylcholine-modified N-glycans were also detected in the triple mutant (Fig. 4). Only residual amounts of glycans lacking the α1,6-mannose (late-eluting GalFuc-modified isomers of *m/z* 1135 and 1338) were observed.

A comparison of HPLC chromatograms of N2 and *aman-3* single mutant demonstrated shifts of major peaks (Fig. S1). Tri- and tetra-fucosylated structures were observed between 10 and 14 min (5.5–7.0 g.u.). While tetra-fucosylated glycans were observed in N2 (Hex_4-6_HexNAc_2_Fuc_4_Me_0-1_), these were completely absent in tm5400 and tri-fucosylated structures (Hex_4-7_HexNAc_2_Fuc_3_Me_0-1_) were more pronounced in this strain (peak *a*, *b* and *i* to *v* in Fig. S1). In comparison to N2, two peaks containing primarily Man_3_GlcNAc_2_, Man_5_GlcNAc_2_, and Man_3_GlcNAc_2_Fuc_1_ structures were decreased in tm5400 (peak *c* and *d* in Fig. S1).

### *In vitro* enzymatic activity and intracellular localization of AMAN-3

**Figure 5:**
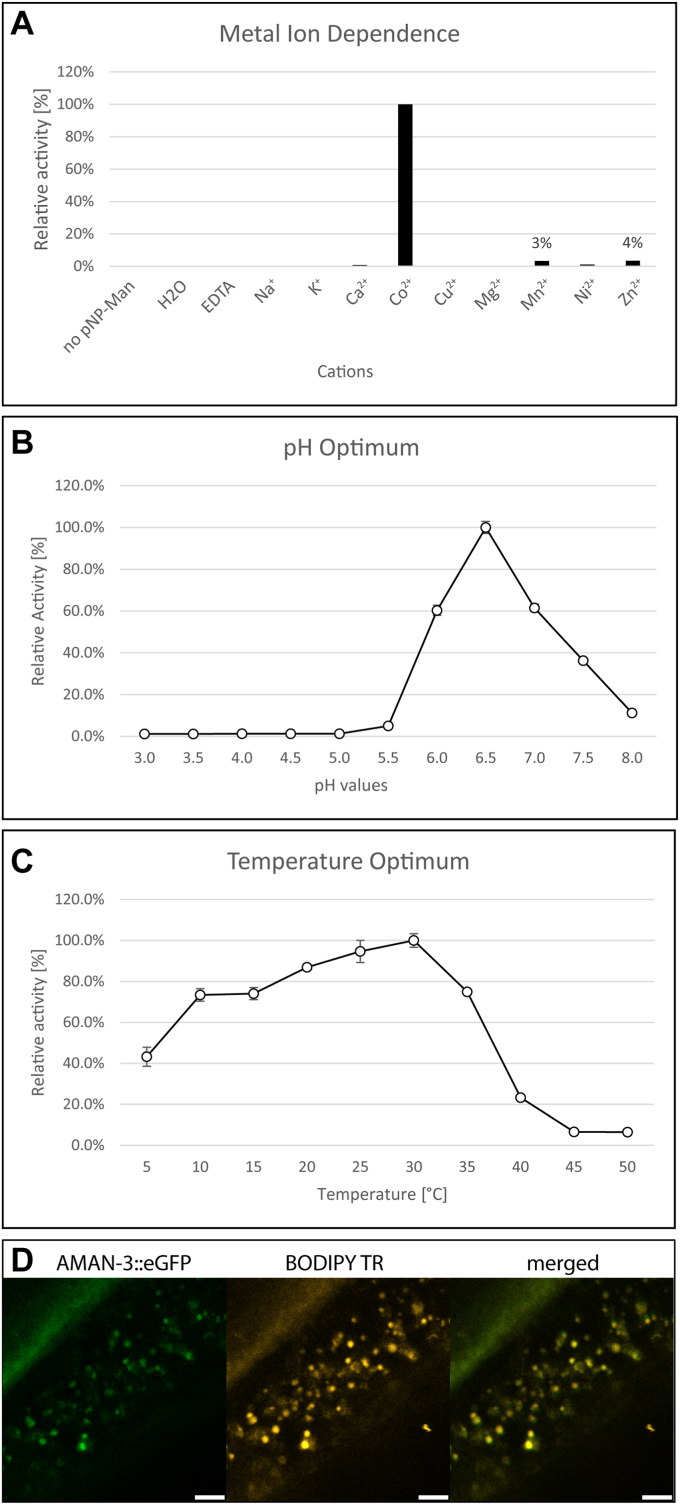
**Enzymatic characterization of recombinant AMAN-3 and confocal micrographs of AMAN-3::eGFP worm.** Analyses were performed in 96-well microtiter plates by incubating purified AMAN-3 with pNP-α-Man under various conditions. OD405 absorption of the reaction mixtures was measured, and relative activities were calculated by comparing obtained values with the highest value in each experiment. Impact of different metal cations on AMAN-3 was investigated, which indicated that CoCl_2_ is a strong activator of AMAN-3 (*A*). The pH optimum of AMAN-3 is at 6.5 (*B*) and temperature optimum is at 30 °C (*C*). Values represent averages ± standard errors (*n* = 4). *D*, confocal images of a paralyzed adult worm demonstrate the overlapped AMAN-3::eGFP signal (*green*) and BODIPY signal (used to stain Golgi apparatus, shown in *gold*). *White bars* at the bottom indicate the length of 5 μm. eGFP, enhanced green fluorescent protein.

The impact of deleting *aman-3* on the N-glycome, especially in the *hex-2;hex-3* background, directed us to more specifically examine the biochemical characteristics of the recombinant AMAN-3, which was expressed in Sf9 cells. His-tagged recombinant AMAN-3 was purified and its sequence was verified by LC-MS/MS (Table S1). The Co(II)-dependence and slightly acidic pH optimum (6.5) were confirmed (Fig. 5, *A* and *B*); AMAN-3 retained over 70% activity in a broad temperature range between 10 °C and 30 °C, whereas the activity quickly declined at temperatures above 30 °C (Fig. 5*C*). The pH optimum data as well as glycomic data strongly suggest that AMAN-3 is a Golgi-resident enzyme. To verify this, confocal microscopy was used to examine the distribution of GFP-fused AMAN-3 in live *aman-3::egfp* worms. Despite the low expression of AMAN-3, micrographs indicated that AMAN-3 signals tend to overlap with fluorescent signals of BODIPY TR ceramide, a dye used to stain Golgi apparatus (Fig. 5*D*).

**Figure 6:**
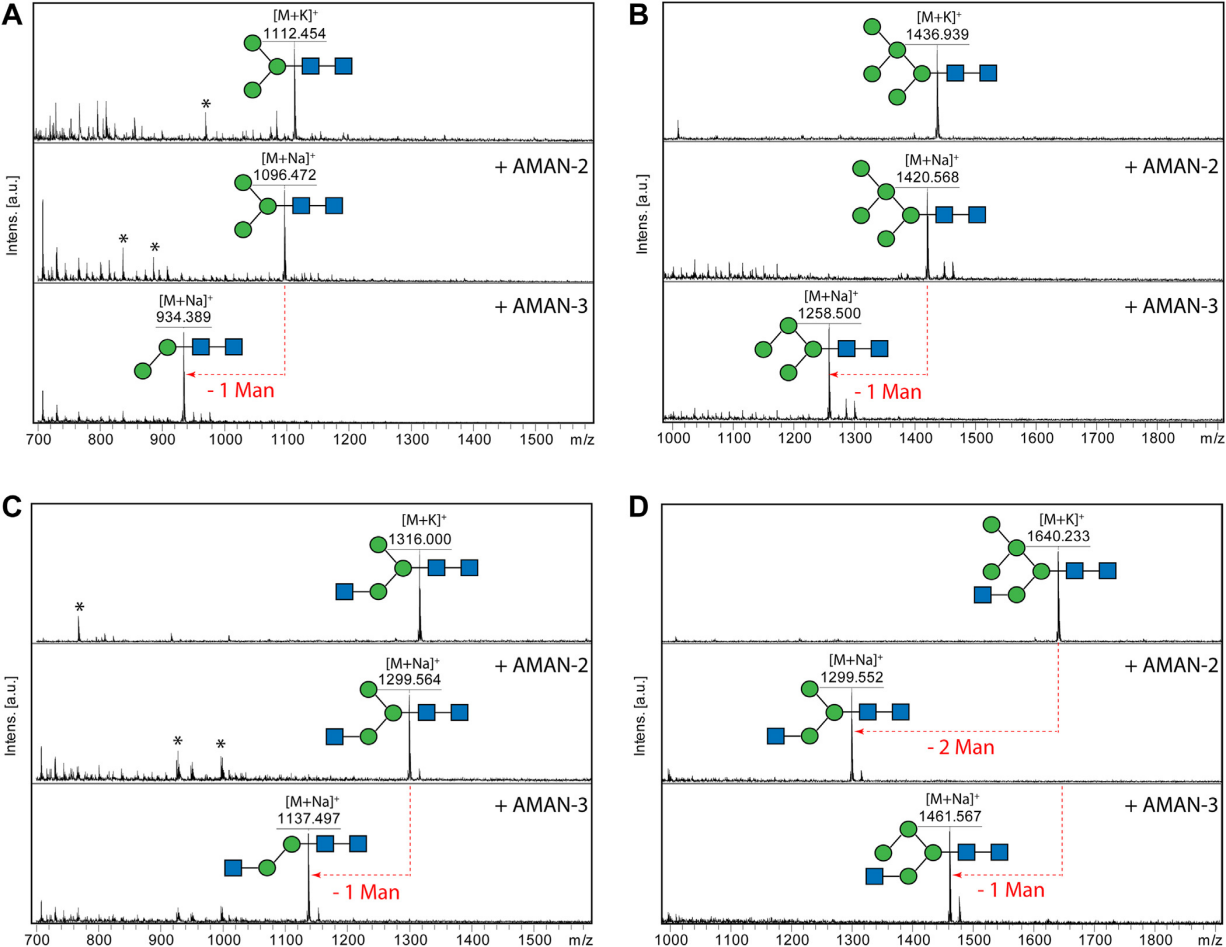
**A comparison of substrate specificities between AMAN-2 and AMAN-3.** Four RP-HPLC purified AEAB-labeled N-glycans (Man_3-5_GlcNAc_2-3_) were incubated with the recombinant mannosidases for 24 h under their optimal conditions. Reaction mixtures were measured by MALDI-TOF MS and MS/MS. The two paucimannosidic structures Man_3_GlcNAc_2_ (*A*, *m/z* 1112) and Man_5_GlcNAc_2_ (*B*, *m/z* 1436) as well as a truncated complex structure Man_3_GlcNAc_3_ (*C*, *m/z* 1316) are substrates of AMAN-3 but not AMAN-2, as indicated by the loss of solely the α1,6-linked mannose residue. AMAN-2 removes two mannose residues from the hybrid N-glycan Man_5_GlcNAc_3_ (*D*, *m/z* 1640), whereas AMAN-3 selectively removes the terminal α1,6-linked mannose from it. Peaks marked with *asterisks* are nonglycan impurities. AEAB, 2-amino-N-(2-aminoethyl)benzamide; GlcNAc, *N*-acetylglucosamine; Man, mannose.

**Figure 7:**
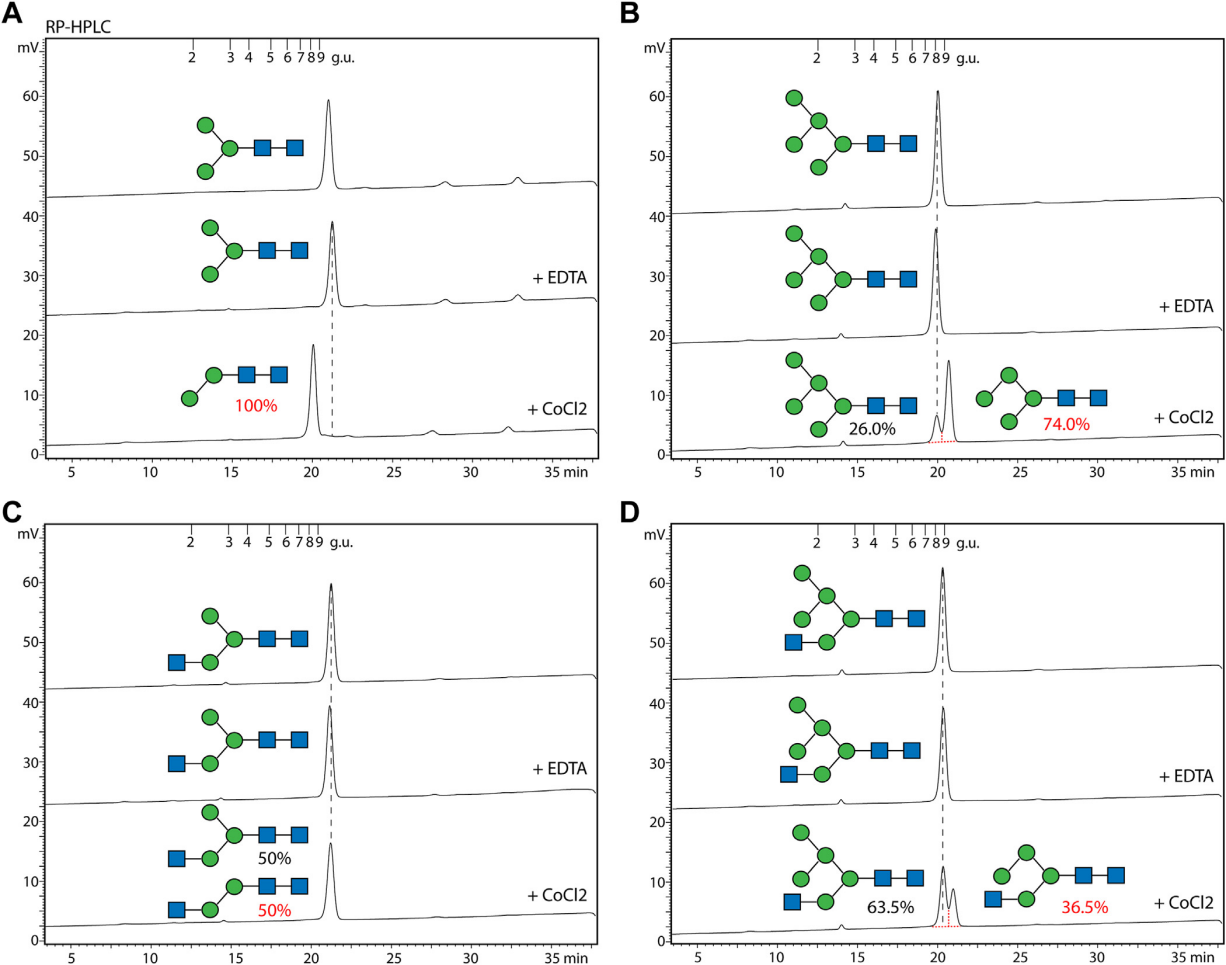
**RP-HPLC quantification of AMAN-3 products.** Equal amount of AEAB-labeled glycans (10 pmol each) were incubated with AMAN-3 in the presence of either EDTA or CoCl_2_. Post 16-h incubation, all samples were heat-inactivated and analyzed by HPLC on the same day. Peak areas were integrated and manually collected fractions were subject to MALDI-TOF MS analysis. Losses of one α1,6-linked mannose from Man_3_GlcNAc_2_ (*A*), Man_5_GlcNAc_2_ (*B*), and Man_5_GlcNAc_3_ (*D*) resulted minor shifts in retention times on the chromatograms. A partial conversion of Man_3_GlcNAc_3_ (*C*) to Man_2_GlcNAc_3_ was confirmed by MS data (Fig. S5). AEAB, 2-amino-N-(2-aminoethyl)benzamide; GlcNAc, *N*-acetylglucosamine; Man, mannose; MS, mass spectrometry.

In terms of substrate specificity, we assayed the classical Golgi AMAN-2 and novel AMAN-3 using HPLC purified N-glycans with defined structures. Only AMAN-3 removed one mannose residue from Man_3_GlcNAc_2_, Man_5_GlcNAc_2_, Man_3_GlcNAc_3_, and Man_5_GlcNAc_3_ as indicated by a mass shift of 162, whereas AMAN-2 only digested Man_5_GlcNAc_3_, removing two mannose residues (Figs. 6 and S2). To further investigate which substrate is favored by AMAN-3, we set reactions at room temperature with equal amount of substrate and quantified the products on HPLC. Data indicated that AMAN-3 removed solely the α1,6-mannose residue from these glycan substrates, which except for Man_3_GlcNAc_3_, resulted in shifts in HPLC retention time (Fig. 7). Full conversion was observed on Man_3_GlcNAc_2_, whereas for Man_5_GlcNAc_2_, Man_3_GlcNAc_3_ and Man_5_GlcNAc_3_ partial conversions (74.0%, 50.0%, and 36.5%, respectively) were observed (Fig. 7). The partial demannosylation from Man_3_GlcNAc_3_ to Man_2_GlcNAc_3_ by AMAN-3 was verified by MALDI-TOF MS and MS/MS (Fig. S3).

### Glycan remodeling using *Caenorhabditis* glycoenzymes

**Figure 8:**
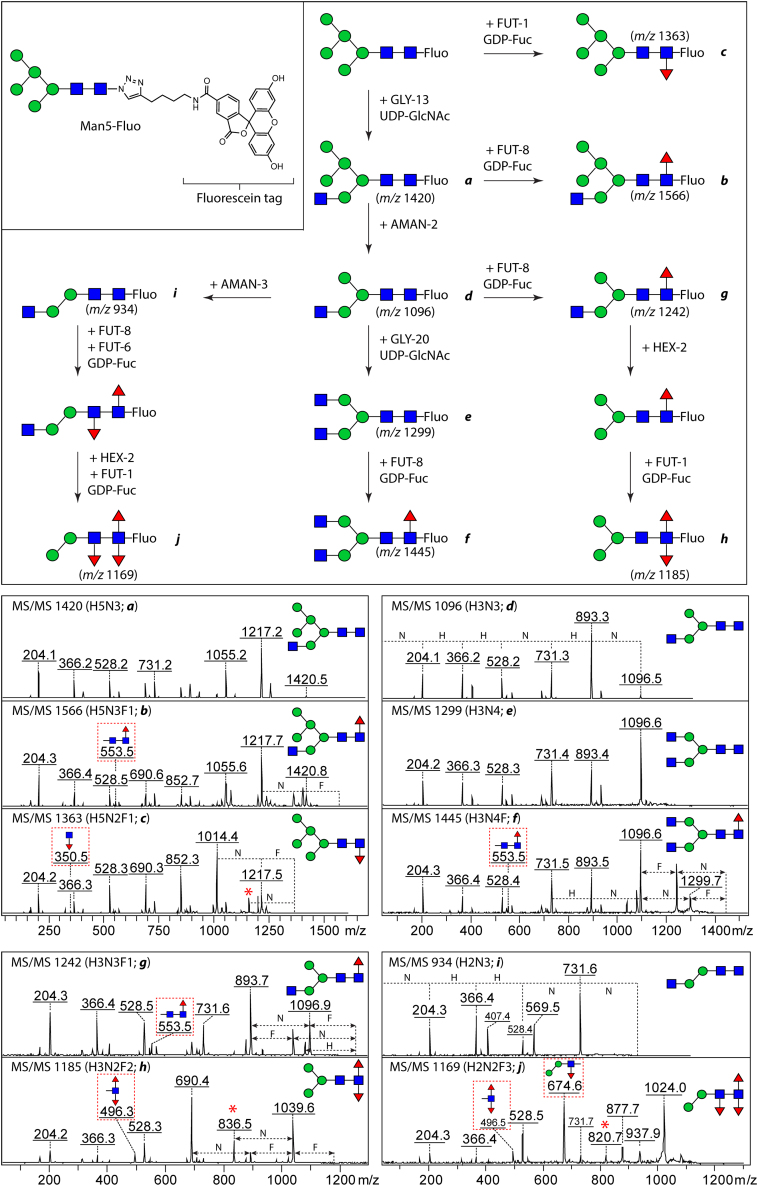
**Remodeling of fluorescein-conjugated N-glycans and MALDI-TOF MS/MS spectra of products.** The remodeling started with a Man_5_GlcNAc_2_ structure; 5 *Caenorhabditis elegans* glycosyltransferases as well as three glycosidases, recombinantly expressed in *Pichia pastoris*, were sequentially used to modify their substrates; afterward, aliquots of the reaction mixtures were analyzed by mass spectrometry. A range of hybrid, complex biantennary, and fucosylated paucimannosidic N-glycan structures were successfully synthesized. Structures of enzymatic products (a to j, *left panel*) were confirmed based on the fragmentation patterns of their parent ions ([M+H-linker]^+^), as well as the knowledge of the substrate specificities of the employed enzymes. Key fragments indicating where fucose is attached were highlighted in *dashed boxes* in *red*; whereas fragment ions, indicative of the “re-arrangement” of fucose residues, were marked with *red asterisks* (*e.g.*, *m/z* 836.5).

The N-glycan biosynthesis in *C. elegans* is a series of very complicated reactions occurring in the endoplasmic reticulum and the Golgi, resulting in a large variety of structures on properly folded glycoproteins (32). Although not yet fully understood, some of the biosynthetic pathways could be deduced from N-glycomics data obtained from WT and KO strains as well as from *in vitro* enzymatic data (33), whereby removal of the α1,6-mannose to result in FUT-6 substrates was considered a missing link. For the first time, we used recombinant glycosyltransferases (GLY-13, GLY-20, FUT-1, FUT-6, and FUT-8) and glycosidases (AMAN-2, AMAN-3, and HEX-2), all recombinant forms of *C. elegans* enzymes, to mimic some of the biosynthetic reactions occurring in the Golgi by remodeling fluorescein containing glycoconjugates (Fig. 8).

Unlike PA-labeled and 2-amino-N-(2-aminoethyl)benzamide (AEAB)-labeled glycans, fluorescein-conjugated glycans have a tendency for in source fragmentation in MS mode; nevertheless, a portion of the intact compounds remained visible on the MS spectra. This is exemplified by analyzing the intact Man5-Fluo glycan (Fig. S4). The MS/MS spectra of [M+H-linker]^+^ ions with a mass difference of 520 Da (loss of the linker due to in-source degradation) are, however, more informative than the spectra of the intact compounds detected as [M+Na]^+^. Therefore, [M+H-linker]^+^ ions were selected for structural assignment in MS/MS experiments and these *m/z* values are those mentioned in the following paragraphs.

Firstly, an aliquot of Man5-Fluo (5 nmol) was incubated in the presence of the donor substrate UDP-GlcNAc with GLY-13, one of three known β1,2-*N*-acetylglucosaminyltransferase I (GnT I) isoforms, to yield [Manα1,6(Manα1,3)Manα1,6](GlcNAc β1,2Manα1,3)Manβ1,4GlcNAcβ1,4GlcNAc-Fluo (Man5Gn-Fluo). Full conversion was observed after overnight incubation as judged by the ion of *m/z* 1420, increased by 203 from *m/z* 1217 (Fig. 8*A*); in addition, a portion of the GnT I product was fully fucosylated by a α1,6-fucosyltransferase (FUT-8), which resulted in the formation of a compound with *m/z* 1566 (corresponding to Fig. 8*B*). The core fucosylation has been confirmed by the presence of a daughter ion HexNAc_2_Fuc_1_ of *m/z* 553.5, a diagnostic ion used for other compounds carrying 6-linked core fucose residue. Under the same conditions, a small aliquot of Man5-Fluo was fully fucosylated by FUT-1 to [Manα1,6(Manα1,3)Manα1,6](Manα1,3)Manβ1,4GlcNAcβ1,4(Fucα1,3)GlcNAc-Fluo and in this case its daughter ion HexNAc_1_Fuc_1_ (*m/z* 350.5, Fig. 8*C*) was more pronounced in the MS/MS spectrum. However, *C. elegans* FUT-8 cannot modify Man5-Fluo with a 6-linked core fucose *in vitro*, even though more enzyme was added and longer incubation was carried out, suggesting that this enzyme strictly requires the presence of GlcNAc on the lower arm of the reducing end. Interestingly, the observation of N-glycans with core α1,6-fucose (minor portion) in a triple GlcNAc-TI KO indicated *in vivo* activity of FUT-8 on Man5-carrying glycoproteins (12). This enzymatic characteristic is identical to the human homologous enzyme FUT-8 published recently (34).

Following modification by GLY-13, Man5Gn-Fluo was processed by a Golgi α-mannosidase AMAN-2 without previous purification; a new compound with *m/z* 1096 was observed after 2-h incubation, indicative of a full conversion to Manα1,6(GlcNAcβ1,2Manα1,3)Manβ1,4Glc-NAcβ1,4GlcNAc-Fluo (also known as MGn; Fig. 8*D*). Subsequently, HPLC purified MGn-Fluo was split for further remodeling into two types of N-glycans: biantennary glycans and fucosylated glycans. The former were achieved by sequential incubation with GLY-20 and FUT-8; the two products were verified by MS/MS (*m/z* 1299 and 1455, Fig. 8, *E* and *F*). A difucosylated glycan was created by serial incubation of MGn-Fluo first with FUT-8 to introduce 6-linked fucose and then with a mixture of HEX-2 and FUT-1 to introduce a 3-linked fucose (Fig. 8, *G* and *H*). The final product carries Manα1,6(Manα1,3)Manβ1,4Glc-NAcβ1,4(Fucα1,6)(Fucα1,3)GlcNAc-Fluo (also called MMF^3^F^6^), which is a typical difucosylated N-glycan occurring in both nematodes and insects (7, 35); this structure was concluded due to a range of daughter ions including *m/z* 496.3 Y-ion and its corresponding B-ion of *m/z* 690.2, suggesting a difucosylation on the inner most nonreducing GlcNAc residue.

In addition, by removing the “upper arm” from MGn-Fluo with AMAN-3, a FUT-6 acceptable substrate was formed (Fig. 8*I*). After sequential modification with core FUTs and HEX-2, removing the nonreducing GlcNAc residue, a trifucosylated glycan Manα1,3Manβ1,4(Fucα1,3)GlcNAcβ1,4(Fucα1,6) (Fucα1,3)GlcNAc-Fluo was achieved as judged by the presence of fragments at *m/z* 496.5 and 674.6 (Fig. 8*J*).

These results provided additional enzymatic data to support the sequential modifications of N-glycans by Golgi resident-glycoenzymes toward the formation of hybrid-, biantennary-, and fucosylated paucimannosidic N-glycans.

### Loss of glycosylation enzymes delays development, reduces animal size, and impairs food-dependent behaviors

**Figure 9:**
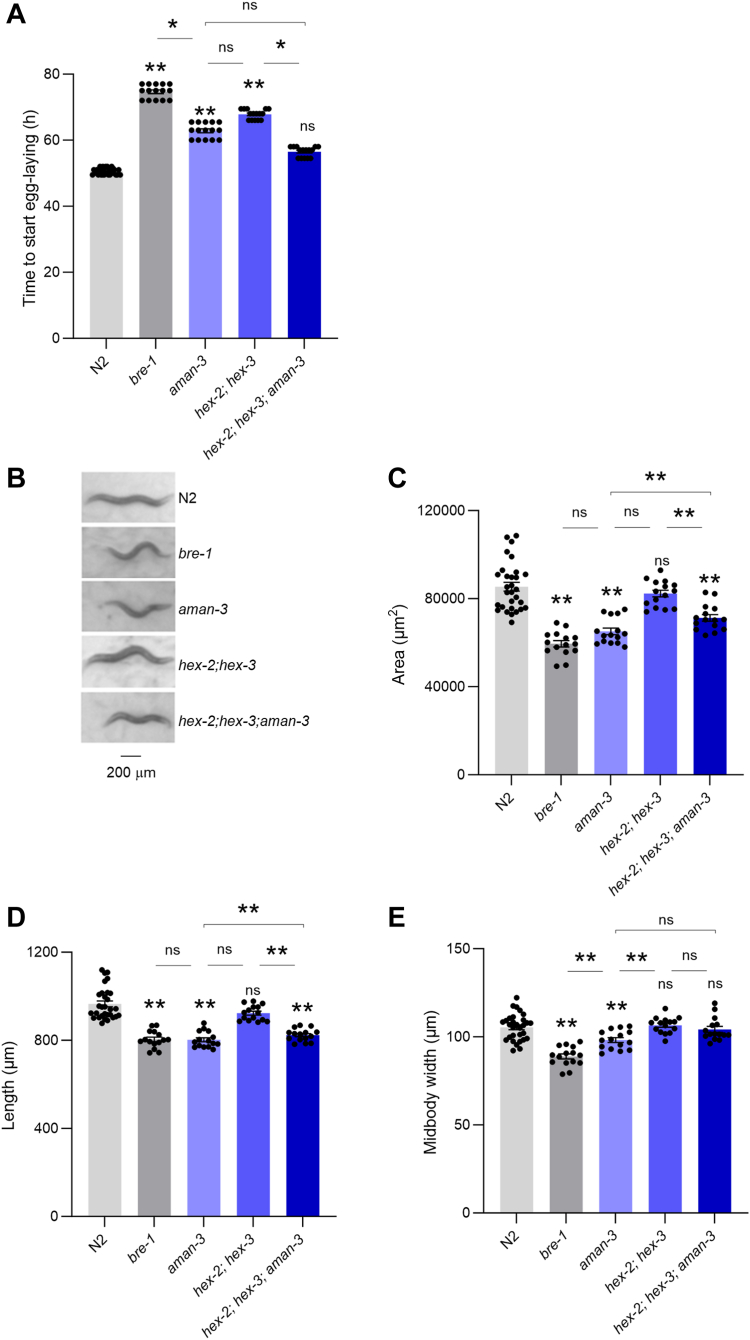
**Growth and morphological defects in glyco-mutants.** Comparison of developmental time (*A*) and adult animal size (*B–E*) between WT (N2) and indicated glyco-mutants grown at 25 °C. Average (bars) ± s.e.m. (error bars) of *n* = 15 assays per genotype, each assay scoring at least 20 animals (*A*, *C–E*). Representative picture of adult worms illustrating size differences (*B*). ∗∗*p* < 0.01; ∗*p* < 0.05, ns, not significant by Dunn (*A*) or Bonferroni (*C–E*) *post hoc* tests. Signs just above the glyco-mutant bars indicate significance levels *versus* N2.

The phenotypic characterization of *C. elegans* glyco-mutants used in this study offer an opportunity to further define the biological role of N-glycan modifying enzymes *in vivo*. All homozygous strains were viable under laboratory conditions and naked eye observation with a regular stereo microscope did not show obvious phenotypes. We thus turned to quantitative assessments of worm growth speed, size, posture, and locomotion. First, we measured the developmental speed by comparing the time from hatching to first egg-laying in WT (N2), *bre-1*, *aman-3*, as well as *hex-2;hex-3* double mutants, and *hex-2;hex-3;aman-3* triple mutants (Fig. 9, *A* and *B*). We found that *bre-1*, *aman-3*, and *hex-2;hex-3* developed significantly slower than WT (Fig. 9*A*). Interestingly, the *hex-2;hex-3;aman-3* triple mutant growth was similar to that of N2 and significantly faster than that of either *aman-3* or *hex-2;hex-3*, suggesting that these mutations genetically interact to modulate developmental speed.

Second, we quantified the size of adult animals (Fig. 9, *B*–*E*). Whereas the size of *hex-2;hex-3* double mutants was similar to that of WT, it was significantly reduced in *bre-1, aman-3*, and *hex-2;hex-3;aman-3* triple mutants. Both animal length and midbody width were reduced in *bre-1* and *aman-3*, whereas only length was reduced in *hex-2;hex-3;aman-3* triple mutants (Fig. 9, *D* and *E*).

**Figure 10:**
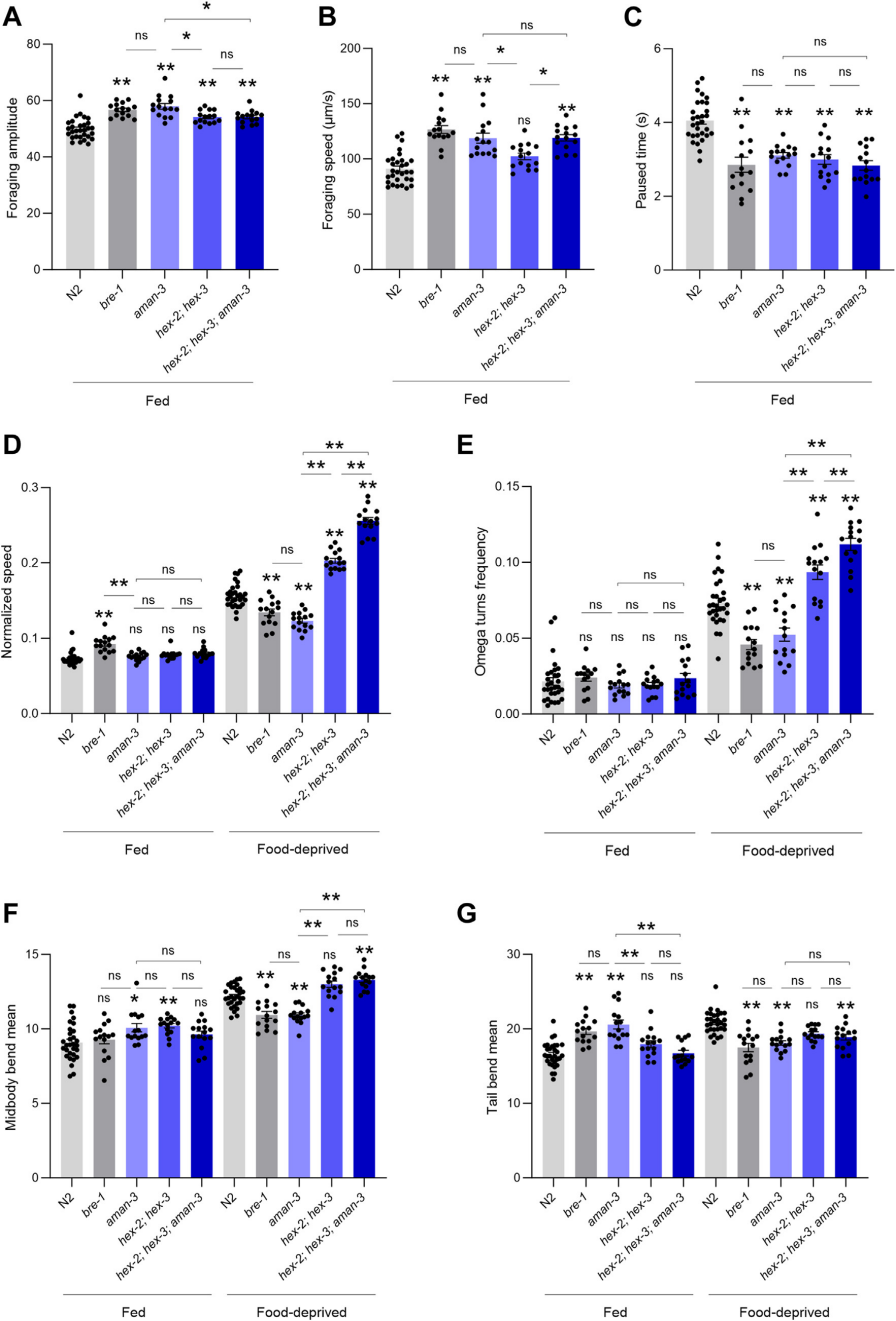
**Postural and locomotion behavior alterations in glyco-mutants.** Comparison of postural and locomotion parameters in adult animals between WT (N2) and indicated glyco-mutants grown at 25 °C and assessed on food (Fed, *A–G*) or assessed off-food 3 h after food deprivation (Food-deprived, *D–G*). Average (bars) ± s.e.m. (error bars) of *n* = 15 assays per genotype, each assay scoring at least 20 animals. ∗∗*p* < 0.01; ∗*p* < 0.05, ns, not significant by Bonferroni (*A–G*) *post hoc* tests. Signs just above the glyco-mutant bars indicate significance levels *versus* N2.

**Figure 11:**
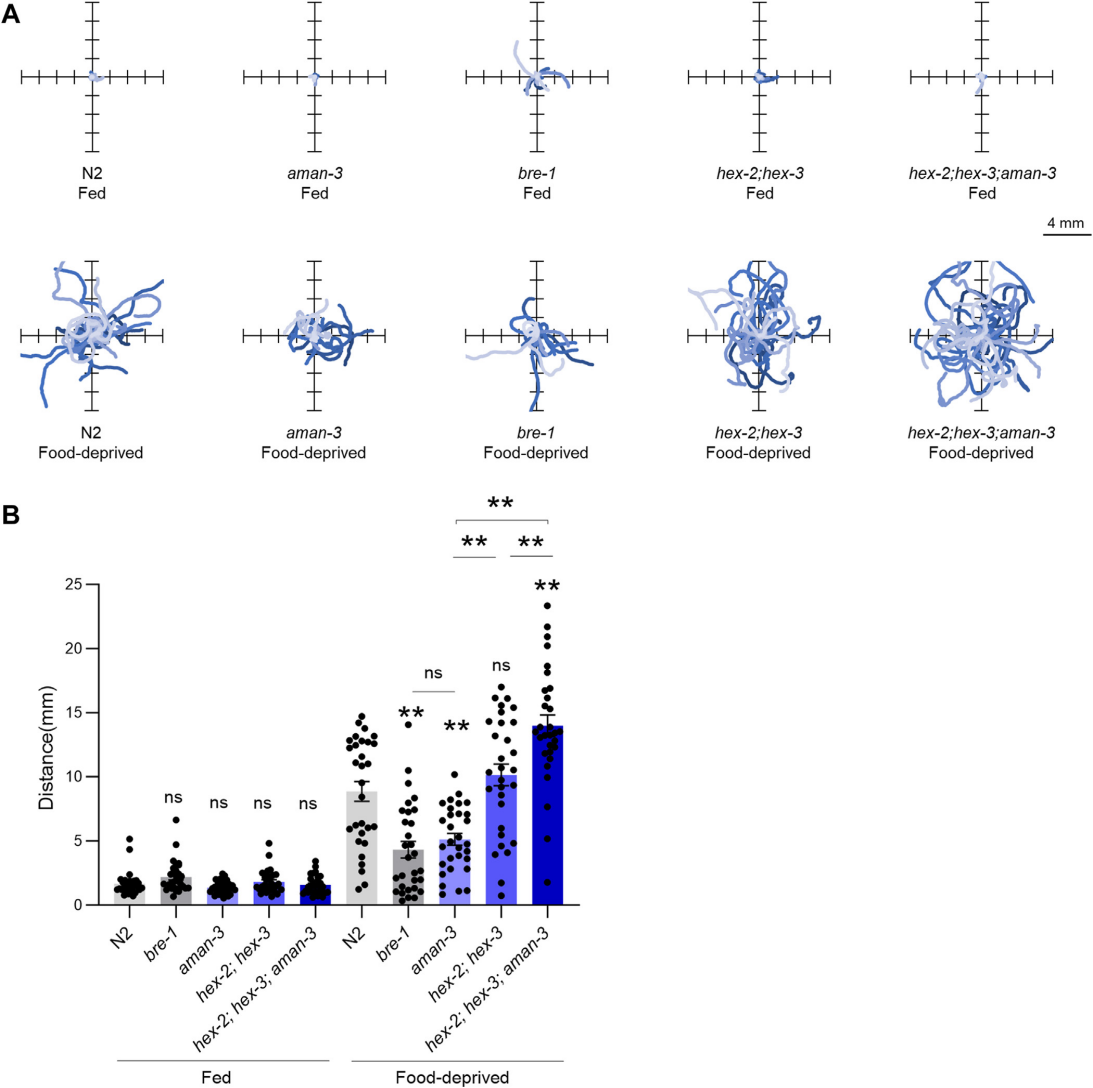
**Alteration of food-search trajectories in glyco-mutants.** One-minute worm trajectories of well-fed worms on food (*A*, *top panels*) and 3 h food-deprived worms off-food (*A*, *bottom panels*). Thirty trajectories per condition plotted from a single starting (0,0) coordinate and illustrating the worm dispersal. Average ± s.e.m. and individual data points for the covered distance (*B*, corresponding to the path length). *n* = 30 animals. ∗*p* < 0.05 and ∗∗*p* < 0.01 by Bonferroni post hoc tests. Signs just above the glyco-mutant bars indicate significance levels *versus* N2.

Third, we used high-content computer-assisted behavioral analysis tools to reveal postural and locomotion differences across strains in crawling animals under fed and starved conditions. On food, WT animals are in a dwelling state (36, 37), characterized by low locomotion speed, frequent pausing, and foraging head movement (side-to-side nose swipes). All four mutant strains displayed altered dwelling behavior, with upregulated foraging movements and downregulated pausing frequency (Fig. 10, *A–C*). As in previous studies (37), food-deprivation caused WT worms to shift to a food-search behavior characterized by increased speed (Fig. 10*D*) and more frequent reorientation events (called omega turns, Fig. 10*E*), hence producing specific dispersal trajectories (Fig. 11*A*), favoring longer-range exploration (Fig. 11*B*). This behavioral state is also associated with a distinct posture, including increased midbody and tail bending (Fig. 10, *F* and *G*). We found that, upon food deprivation, the *bre-1* mutants and *aman-3* mutants displayed reduced speed, omega turn frequency, midbody, and tail bending as compared to WT (Fig. 10, *D*–*G*). Examples of typical postures with reduced curvature are presented in Fig. S5. In contrast, *hex-2;hex-3* double mutants had an opposite phenotype with increased speed and omega turn frequency (Fig. 10, *D* and *E*). The triple *hex-2;hex-3;aman-3* mutant displayed even further increase in speed and omega-turn frequency, with values significantly increased in comparison to all the other genotypes. These specific behavioral differences across genotypes were associated with different trajectories and dispersal efficacy, that is likely relevant for food-search behavior (Fig. 11).

Collectively, our phenotypic data in glyco-mutants highlights a role for *aman-3, hex-2/3* and *bre-1* in controlling worm developmental speed, animal size, dwelling behavior on food and food-search behavior in response to food-deprivation. Furthermore, the various phenotype-specific genetic interaction types between *aman-3* and *hex-2;hex-3* mutations suggest that the complex, nonadditive impact that the losses of AMAN-3 and the two hexosaminidases have on N-glycan structures translates into pleiotropic phenotypic impact.

## Discussion

Probably all multicellular eukaryotes have multiple members of the so-called GH38 α-mannosidase family. At least one is lysosomal or vacuolar and involved in glycoconjugate degradation, whereas one or more have roles in N-glycan remodeling in the Golgi apparatus (38). In a previous study, the *C. elegans* AMAN-3 was identified as a homolog of the Golgi α-mannosidase II (AMAN-2) and the nonpurified recombinant enzyme showed to react with pNP-α-mannoside, an artificial substrate (23). Here, we confirm and expand on these previous observations to substantiate the notion that AMAN-3 is a Golgi-resident α1,6-mannosidase that uses N-glycans as substrates, representing a sought-after missing link in the N-glycan biosynthetic pathway of *C. elegans* and a number of related nematode species, and filling an important gap in our understanding of the complex of enzymatic reactions.

AMAN-3 fits the characteristics of typical Golgi-resident mannosidases, which possess N-terminal transmembrane domains, are active at a slightly acidic pH and require metal ions as cofactors (23). Our GFP-fusion confocal data in this study are also in line with a Golgi localization of AMAN-3. Interestingly, AMAN-3 activity depends on the presence of Co(II) as divalent metal ion. This feature is very similar to a *Spodoptera frugiperda* Golgi α-mannosidase, the SfMANIII, as well as the *Drosophila* Man-IIb (Table 1). Both insect and nematode enzymes can be inhibited by swainsonine, a common GH38 α-mannosidase inhibitor (21, 22). Other Co (II)-dependent mannosidases include cytosolic or lysosomal enzymes with roles in degradation, such as mammalian MAN2C1 and MAN2B2 (39, 40).

**Table 1:** A comparison of insect and nematode Golgi α-mannosidase III

Enzymes	SfManIII	Dm ManIIb	Ce AMAN-3
Characteristics	GnT I-independent	GnT I-independent	GnT I-independent
Optimal pH	6.5	5.8	6.5
Optimal temperature	n.a.	37 °C	30 °C; >70% activity at 10–30 °C
Inhibitor	Swainsonine	Swainsonine	Swainsonine
Substrates	Man_6-9_GlcNAc_2_	Man_8-9_GlcNAc_2_	Man_3_GlcNAc_2_
	Man_5_GlcNAc_2_	Man_5_GlcNAc_2_	Man_5_GlcNAc_2_
			Man_3_GlcNAc_3_
			Man_5_GlcNAc_3_
Cobalt activation	Yes	Yes	Yes
Specificity	α1,2-linked mannose	α1,2-linked mannose	α1,6-linked mannose
	α1,3/6-linked mannose	α1,3/6-linked mannose	
Reference	Kawar *et al.* (21)	Nemčovičová *et al.* (22)	Paschinger *et al.* (23) and data in this study

**Figure 12:**
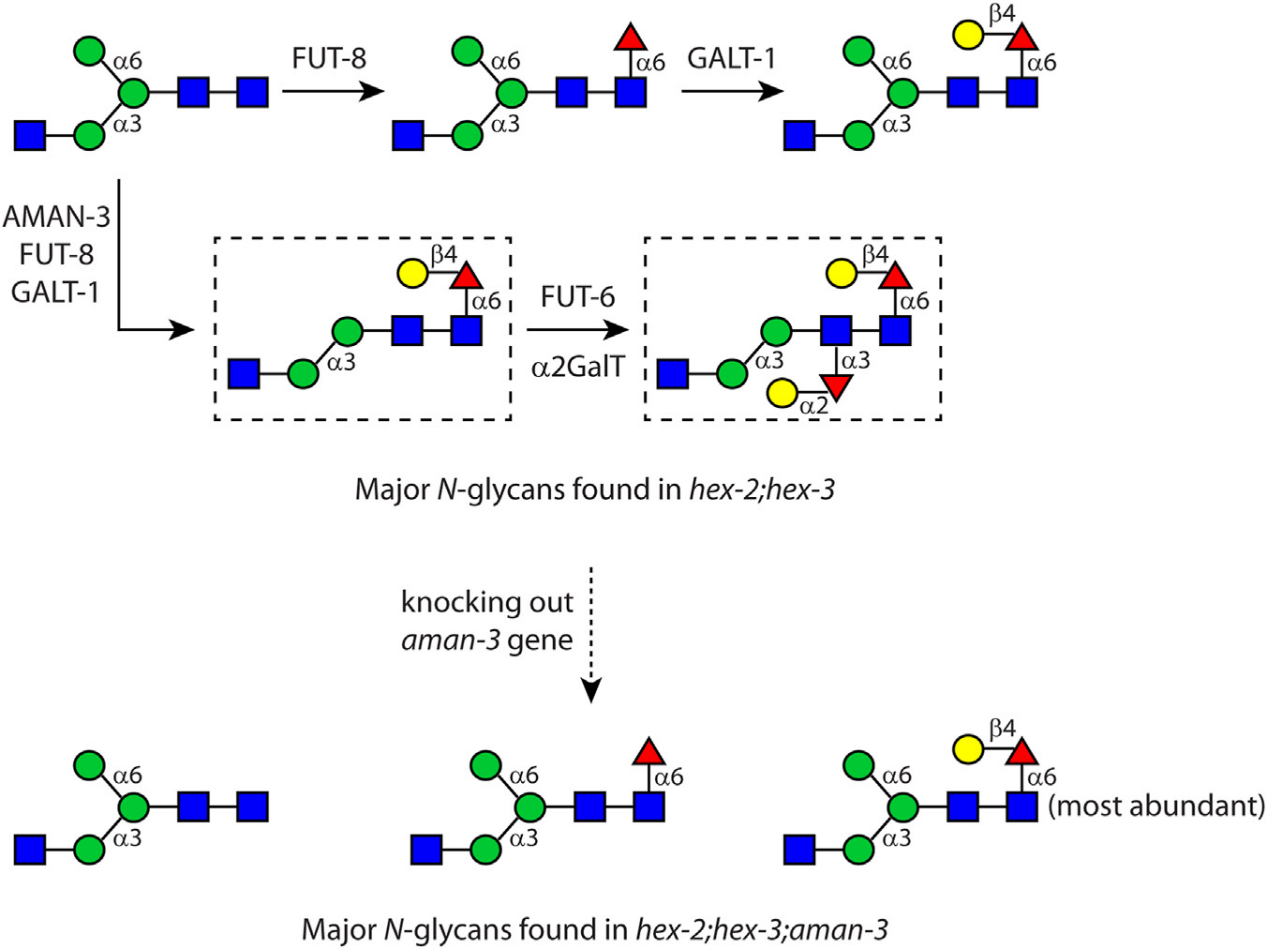
**A summary of major N-glycan structures found in the double (*hex-2;hex-3*) and in the triple (*hex-2;hex-3;aman-3*) mutants.** Enzymes that participate relevant biosynthetic pathways are annotated. Glycans without the “upper arm” (shown in dashed boxes), abundant in *hex-2;hex-3*, are abolished after knocking out the *aman-3* gene.

In this study, we used reverse genetics and comparative glycomics to obtain a very detailed view of the impact of *aman-3* loss, and combined these approaches with a comprehensive *in vitro* reconstitution system of glycan remodeling able to reveal AMAN-3 action in the context of a complex substrate pool. Our glycomics data provided direct evidence that the *aman-3* gene is essential to the biosynthesis of tetra-fucosylated N-glycans occurring in the N2 WT worms. Knocking out *aman-3* in *hex-2;hex-3* double mutant resulted in an even more dramatic shift in the N-glycome, as judged by the absence of fucose modification on the distal GlcNAc residue. One signature glycan structure in *hex-2;hex-3* with a composition of Man_2_GlcNAc_3_Fuc_2_Gal_2_ is completely abolished (Fig. 12). Collectively, our data indicated that AMAN-3 plays a key role in removing a “block” to modification by FUT-6 of the distal (second) core GlcNAc residue. Thus, it can be surmised that orthologues of AMAN-3 work in concert with orthologues of FUT-6 in species such as *H. contortus*, *O. dentatum*, and *A. suum*, but that these are absent from species such as *D. immitis* or *T. suis* which lack trifucosylated core chitobiose motifs (9, 20, 41). The biological role of these structures in the context of parasite-host interactions remains unclear. However, it is possible that mammalian hosts recognize these structures of parasites, as they significantly differ from mammalian glycans. Indeed, the question as to how the natural processing of parasite antigens affects their immunogenicity as well as their ability to act as protective epitopes is still open. Studies on *H. contortus* H11 antigens have indicated that recombinant forms do not induce protective immunity, even if expressed in *C. elegans* (42); however, the ability to better reproduce natural *H. contortus* glycosylation in recombinant expression systems by using AMAN-3 and FUT-6 may bring us closer to understanding the interplay between nematode glycosylation and host immune systems.

Beyond its likely relevance for parasite-host interaction, our study provides direct insight on the more general biological importance of AMAN-3, BRE-1, and HEX-2/3 enzymes in *C. elegans*, including their roles in growth and behavior modulation. Interestingly, our data show that the different mutations produce sometime similar and sometimes opposite impact on the different phenotypic parameters. For instance, *bre-1* and *hex-2/-3* mutations both reduce growth speed, but their respective impact on animal speed and omega turn during food search was opposite. Likewise, the epistatic analysis between *aman-3* and *hex-2;hex-3* showed different synthetic effects, with positive epistasis effects for food-search behavior parameters, but a negative epistasis effect for development speed. These complex phenotypic patterns are not very surprising in the light of the complex glycome alterations that these mutations produce, which are not “simply additive”. Indeed, each mutant produces a unique pattern of N-glycans, missing some WT structures and harboring some abnormal structures (Figs. 3 and 4). Behavioral differences observed in worm glyco-mutants demonstrated a close connection between protein glycosylation and developmental defects as exemplified by studies on *aman-2* and *cogc* mutants (43, 44), which is also well-known in human congenital disorders of glycosylation (45). Our approach combining glycome data with high-content phenotypic characterization paves the road for future studies with additional *C. elegans* glyco-mutants in order to get a more comprehensive and systematic understanding of the relationships between glycome changes and their phenotypic impact in this powerful genetic model.

## Experimental procedures

### Preparation and cultivation of *C. elegans* strains

The N2 WT *C. elegans* and HY496 mutant (*bre-1* deficiency) were obtained from the *Caenorhabditis* Genetics Center, University of Minnesota, MN and an *aman-3(tm5400)* single mutant, carrying a 401 bp deletion and 2 bp insertion in the *aman-3* gene, was obtained from the National Bioresource Project for the Experimental Animal Nematode *C. elegans*, Tokyo Women’s Medical University, Japan. A *hex-2;hex-3* double mutant (16) was previously prepared by crossing the single *hex-2(tm2530)* mutant and *hex-3(tm2725)* mutant, which were obtained through the National Bioresource Project; this double mutant was used to prepare a *hex-2;hex-3;aman-3* triple mutant (cop1842) using the CRISPR/Cas9 approach by Knudra Transgenics (NemaMetrix). Using two *aman-3*-targeting sgRNAs to guide Cas9, a 5145-bp fragment was deleted in the genome of the *hex-2;hex-3* mutant and replaced with a 18-bp insertion containing a 3-frame stop sequence (5′-TAAATAAATAAACTCGAG-3′). Screening was carried out by genotyping PCR using primer pairs A (5′-AGCCTCAATTTCGTCTACACCAC-3′ and 5′-GGGCAAATCGGAGTCATCTGAA-3′, 652 bp as the WT) and B (5′-AGCCTCAATTTCGTCTACACCAC-3′ and 5′-CCTGTTTTGCACCCAGTTTGATG-3′, 724 bp as *aman-3* KO, Fig. S6) and the deletion of *aman-3* gene was confirmed by DNA sequencing. Moreover, an enhanced green fluorescent protein (eGFP)-encoding DNA fragment was fused to the *aman-3* gene of N2 worms using CRISPR/Cas9 technology. This knock-in strain (*aman-3::egfp,* strain name PHX5021; allele *syb5021*) was custom-made by SunyBiotech.

All worm strains were cultured under standard conditions at 20 °C. For a large-scale preparation, worms were grown in S-complete medium supplied with *Escherichia coli* OP50 in shake flasks and were purified by sucrose density centrifugation; after intensive washing in saline solution, worms were stored at −80 °C prior to analysis.

### Mannosidase activity assays using worm lysate

N2, *aman-3*, and *hex-2;hex-3* worms were harvested from nematode growth medium agar plates and repeatedly washed in saline solution (0.9% NaCl) to remove OP50. Worms were transformed in a tissue grinder and homogenized in 300 μl of lysis buffer containing 20 mM Mes buffer, pH 6.9, 0.5% Triton X-100, and 0.01% protease inhibitor cocktail (Sigma-Aldrich); post centrifugation to remove cell debris (14,000*g* for 4 min at 4 °C) clear supernatants of the worm lysates were kept for enzymatic assays. Reaction mixtures were prepared by mixing 1 μl of PA-MM substrate (2D-HPLC purified) and 0.8 μl of worm supernatant with 25 mM ammonium acetate buffer (pH 6.5) supplied with or without 6 mM cobalt (II) chloride (CoCl_2_) or 0.6 μM Swainsonine (Sigma-Aldrich). Reactions were incubated at room temperature prior to HPLC analysis.

### Confocal microscopy

Worms at mixed developmental stages were harvested and incubated in M9 buffer supplied with 5 μM BODIPY TR Ceramide complexed to bovine serum albumin (Invitrogen) at room temperature (1 h). Prior to immobilization on poly-L-lysine–coated glass slides, worms were thoroughly washed in M9 and paralyzed in 20 mM levamisole hydrochloride (Sigma-Aldrich). Images were recorded using a Leica TCS SP5 laser scanning confocal microscope (Wetzlar) as described previously (27).

### Molecular cloning

Reagents and kits used for molecular cloning are mostly purchased from the New England Biolabs unless specified. DNA oligonucleotides were ordered from Sigma-Aldrich. The *C. elegans aman-2* coding sequence was subcloned from a pPICZαC construct (23) to a reengineered pPICαHisFLAG vector (15) using the Gibson Assembly (GA) approach. GA primers (5′-CGACGATGACAAGCTGCAGGCATCGATGAAAGATGTTTGTGG-3′ and 5′-GCTGGCGGCCGCCCGCGGTTAAAATGATACAAGAATACTG-3′) were used to amplify a truncated *aman-2* gene (encoding aa 126–1145) by two-step PCR using a Q5 HIFI DNA polymerase; DNA fragment was ligated to an empty pPICαHisFLAG vector using NEBuilder HiFi DNA Assembly Cloning Kit and the reaction mixture was transformed to NEB5α competent cells. Followed by PCR screening, constructs of positive clones were DNA sequenced (LGC genomics). The *aman-2* construct was linearized by *Pme I*, purified and transformed by electroporation to the GS115 strain of *Pichia pastoris* for protein expression. (Note that despite previous success with an untagged AMAN-3 (23), an N terminally tagged form was not expressed; thus, insect cell-based expression was employed as described below.)

To express AMAN-3 in insect cells, the truncated *aman-3* gene (encoding aa 35–1046) was PCR amplified (5′-GCAGCCATCAAAGATTAGGACAGCA-3′ and 5′-CGTCGACGTAGGTCATCGATAAAGAATCAA-3′) and ligated in-frame to an engineered pACEBac1 vector (Fig. S7), which contains DNA fragments encoding a melittin signal sequence (MKFLVNVALVFMVVYISYIYA), a His-FLAG tag (HHHHHHDYKDDDDK) and a thrombin cleavage site (LVPRGS) on the N terminus of AMAN-3. This construct is incorporated into the baculovirus genome following the instruction provided with the MultiBac cloning kit (Geneva Biotech).

### Recombinant protein expression and purification

Recombinant forms of *C. elegans* AMAN-2 (with or without a His-tag), AMAN-3 (nontagged) (23), GLY-13 (nontagged), GLY-20 (nontagged) as well as His-tagged HEX-2 (46), FUT-1, FUT-6, and FUT-8 (12) were expressed in *P. pastoris* for 72 to 96 h at 16 °C following the manufacturer’s manual. Methanol was added to the culture daily to maintain an induction concentration at 1%. Harvested culture supernatants were concentrated and buffer-exchanged by ultrafiltration using 30 kDa molecular weight cutoff centrifugal devices (Sartorius). Hand-packed nickel affinity columns (Qiagen) were used to obtain purified recombinant proteins that carry an N-terminal poly-histidine tag (AMAN-2, HEX-2, and FUTs).

His-tagged AMAN-3 was expressed in Sf9 insect cells. Briefly, cells were maintained in HyClone SFM4Insect medium (GE HealthCare) supplied with 3% of fetal bovine serum (Gibco) at 27 °C. Post transfection of Sf9 cells with a Bacmid DNA (2 μg) using 10 μl of FuGene (Promega), recombinant baculoviruses carrying *aman-3* gene were harvested (V_0_) and used to infect Sf9 cells in a 68 ml suspension culture (80 rpm, 27 °C). 3 days post infection, the recombinant AMAN-3 was purified from the cell culture supernatant by His-tag purification. The protein sequence was verified by LC-MS/MS at the MS core facility of the University of Veterinary Medicine Vienna.

### Mannosidase activity assays using recombinant enzymes

To assess the biochemical property of AMAN-3, including metal ion dependency, temperature and pH optima, a colorimetric assay using *p*-nitrophenyl-α-mannopyranoside as a substrate (pNP-Man, dissolved in dimethyl sulfoxide) and Co(II) was employed as previously described (23). Briefly, His-Tag-purified AMAN-3 was incubated with 5 mM pNP-Man in quadruplicate in a 96-well plate under various conditions using McIlvaine buffers. Post terminating the reactions with 250 μl of 0.4 M glycine-NaOH, pH 10.4, absorbance at 405 nm (*A*_405_) were measured using a Tecan Infinite M200 micro-plate reader.

To generate N-glycan substrates for testing recombinant mannosidases, a Man_8_GlcNAc_2_ structure conjugated with *AEAB* (purchased from NatGlycan LLC) was remodeled. Subsequently, 10 nmol of this compound was first digested with α1,2-mannosidase (New England Biolabs; yielding Man_5_GlcNAc_2_) and then processed by GLY-13 in the presence of donor substrate UDP-GlcNAc and Mn (II) (yielding Man_5_GlcNAc_3_). Man_3_GlcNAc_3_ and Man_3_GlcNAc_2_ structure were prepared by digesting Man_5_GlcNAc_3_ with AMAN-2 alone or in combination with HEX-2. All resulted compounds were HPLC purified and quantified based on the integrated areas of corresponding eluent peaks.

### Release of N-glycans and PA-labeling

*C. elegans* strains at mixed-stages (4–6 g in wet weight) were boiled in water to denature endogenous proteases and glycosidases; worms were homogenized in a tissue grinder prior to a 2-h proteolysis at 70 °C in a round-bottom glass flask using thermolysin (Promega, 1 mg per 1 g wet-weight worm) in 50 mM ammonium bicarbonate buffer (pH 8.5) supplied with 0.5 mM CaCl_2_ (25). Post centrifugation to remove insoluble cell debrides, glycopeptides were enriched by cation exchange chromatography (Dowex 50W × 8; elution with 0.5 M ammonium acetate, pH 6.0) and desalted by gel filtration (Sephadex G25, 0.5% acetic acid as solvent). N-linked glycan was released using a recombinant *Oryza sativa* PNGase A (New England Biolabs) in 50 mM ammonium acetate buffer (pH 5.0) overnight at 37 °C. Native glycans were separated from residual peptides by cation exchange chromatography (Dowex 50W × 8); glycans collected in the filtrate fraction (as no longer bund to the column) were further purified using hand-packed C18 cartridges and nPGC cartridges prior to analysis. Native glycans were labeled with 2-aminopyridine (PA) to induce a fluorescent tag at the reducing ends, and the excess reagent was removed by gel filtration (Sephadex G15, 0.5% acetic acid as solvent) as previously described (25).

### Glycan remodeling

In total, eight active enzymes (AMAN-2, AMAN-3, GLY-13, GLY-20, HEX-2, FUT-1, FUT-6, and FUT-8) were used for the remodeling experiments. All recombinant enzymes were expressed in-house as described above. Except for His-tagged enzymes that were purified, crude enzymes post concentration and buffered-exchange against a storage buffer (25 mM Tris–HLC, 150 mM NaCl, pH 7.0) were used. A fluorescein-conjugated Man_5_GlcNAc_2_ structure (Man5-Fluo) was chemically synthesized and verified by NMR (detailed in Supporting information).

In brief, 5 nmol of Man5-Fluo was used as the initial acceptor substrate, sequentially modified by glycosyltransferases and glycosidases according to the experimental design (Fig. 8). For assays using glycosyltransferases, mixtures containing 80 mM MES buffer (pH 6.5), 20 mM MnCl_2_, substrate, 2 mM nucleotide sugar and relevant enzyme were prepared, whereas for assays using glycosidases, mixtures containing 160 mM Mes buffer, substrate and relevant enzyme were prepared. Incubation was carried out overnight at 37 °C to ensure the full conversion, except for AMAN-2 which was incubated for 2 h to reduce a by-product. Reaction mixtures were directly analyzed by MALDI TOF MS, and a few reaction products were HPLC purified prior to MS analyses to obtain improved MS and MS/MS data.

### HPLC methods

A Shimadzu Nexera UPLC system equipped with a RF 20AXS fluorescence detector was used to analyzed and fractionate different glycoconjugates. For N-glycomics studies, 2-aminopyridine (PA)-labeled worm glycans were fractionated over an Ascentis RP-amide column (2.7 μm, 15 cm × 4.6 mm attached to a 5 cm guard column; Sigma-Aldrich) on HPLC. A gradient of 30% (v/v) methanol (buffer B) in 0.1 M ammonium acetate, pH 4.0 (buffer A) was applied at a flow rate of 0.8 ml/min as follows: 1% buffer B per minute over 35 min (excitation/emission: 320 nm/400 nm) (17). In addition, separation of PA-glycans was also achieved using a Hypersil ODS column (Agilent Technologies) with the same buffers and detector settings, but a flow rate of 1.5 ml/min.

A HyperClone reversed phase column (5μ ODS C18, 250 × 4 mm; Phenomenex) was used for the separation and quantification of AEAB-labeled N-glycans and the purification of fluorescein-conjugated glycans. AEAB-glycans: 0.1 M ammonium acetate, pH 4.0 as buffer A and 30% (v/v) methanol as buffer B, and an optimized gradient as follows: 0 to 10 min, 0 to 20% B; 10 to 35 min, 20 to 50% B; 35 to 35.5 min, 50% B; 35.5 to 36 min, 0% B; 36 to 40 min, back to starting conditions. The flow rate was set at 1.5 ml/min with a maximal pressure at 375 bars and the fluorescence detector setting was Ex 330 nm and Em 420 nm. Fluorescein-glycans using 0.1% formic acid as buffer A and a mixture of 99.9% acetonitrile and 0.1% formic acid as buffer B; detector setting was Ex 490 nm and Em 500 nm. All HPLC fractions were manually collected, dried, and further examined by MALDI TOF MS.

### MALDI-TOF MS and MS/MS

Lyophilized glycan samples, either in native or derivatized forms (PA, AEAB, and fluorescein conjugates), were dissolved in HPLC grade water and subject to MALDI-TOF MS and MS/MS analyses on an Autoflex Speed instrument (1000 Hz Smartbeam-II laser, Bruker Daltonics) using 6-aza-2-thiothymine as a matrix (47). Calibration of the instrument was routinely performed using the Bruker Peptide Calibration Standard II to cover the MS range between 700 and 3200 Da. The detector voltage was normally set at 1977 V for MS and 2133 V for MS/MS; typically, 3000 shots from different regions of the sample spots were summed. Automatic measurements were conducted on HPLC-fractionated glycan samples (N2, tm5400, and cop1842) using the AutoXecute (https://www.cmu.edu/chemistry/facilities/cma/instruments/manuals/flexcontrol-user-manual.pdf) feature of the control software (Bruker flexControl 3.4). To obtain MS/MS spectra, parent ions were fragmented by laser-induced dissociation without applying a collision gas (precursor ion selector was generally ±0.6%). For PA and AEAB-labeled glycans, [M+H]^+^ ions were favored for laser-induced dissociation fragmentation, whereas for fluorescein conjugates [M+H-linker]^+^ ions, as “in-source degradation” products of the intact compounds, were fragmented because their MS/MS spectra presented the most informative fragments for structural annotation.

### Growth, morphological, and behavioral analyses

Animal synchronization was made by treating gravid adults with standard hypochlorite-based procedure. Developmental speed was assessed by measuring the time between hatching and the onset of egg laying in animals maintained at 25 °C on regular nematode growth medium plates seeded with *E. coli* OP50. For each genotype, the growth time prior to behavioral assays was adjusted in order to test animals just after they started to lay their first eggs. Animal size, postural, and locomotion parameters were obtained from videos recorded and analyzed as in Thapliyal *et al.* (37) using the Tierpsy Tracker v1.4 (https://github.com/ver228/tierpsy-tracker) (48). Fifteen assays per condition, each tracking a population of at least 20 animals, were recorded and the average value of selected postural and locomotion parameters extracted for each assay. Individual data points (dots) overlaid in the different bar graphs each represent the value derived from one assay. The “food-deprived” condition was acquired 3 h after food deprivation. Locomotion speed was normalized to the body length in order to compensate for variable animal sizes across genotypes. D’Agostino & Pearson test (*p* < 0.01) was used to test normality of distributions. Comparisons giving significant effects (*p* < 0.05) with ANOVAs were followed by Bonferroni post hoc tests. Dunn’s test was used as nonparametric test whenever the normality assumption criterion was not fulfilled. All tests were two-tailed.

## Data availability

Relevant MS and MS/MS data are converted to mzxml files and uploaded to GlycoPost: https://glycopost.glycosmos.org/entry/GPST000395.

## Supporting information

This article contains supporting information (Supplementary Figures 1–7 and Supplementary Table 1).

## Conflict of interest

The authors declare that they have no conflicts of interest with the contents of this article.
