# Supplementary material for: N-glycan core tri-fucosylation requires Golgi α-mannosidase III activity that impacts nematode growth and behavior
Source: J Biol Chem. 2024 Oct 29;300(12):107944. doi: 10.1016/j.jbc.2024.107944 (PMC11697051; doi:10.1016/j.jbc.2024.107944)
Supplement: Supplementary Information [file mmc2.docx]

**Supplementary Information**

N-glycan Core Tri-fucosylation Requires Golgi α-mannosidase III Activity that Impacts Nematode Growth and Behaviour

Jonatan Kendler, Florian Wöls, Saurabh Thapliyal, Elsa Arcalis, Hanna Gabriel, Sascha Kubitschek, Daniel Malzl, Maria R. Strobl, Dieter Palmberger, Thomas Luber, Carlo Unverzagt, Katharina Paschinger, Dominique A. Glauser, Iain B. H. Wilson and Shi Yan

**Further information regarding the glycomic analyses**

***Definition of the level of the glycan structural analysis:***

To compare compositional differences of the overall N-glycomes of *C. elegans* N2 wild type and knockout stains, native N-glycans enzymatically released from worm materials were profiled using MALDI-TOF mass spectrometry. To gain structural details, the N-glycomes of N2, tm5400 and *hex-2;hex-3;aman-3* were subject to HPLC fractionation and MALDI-TOF MS/MS experiments.

***Search parameters and acceptance criteria***

1. **Peak lists:** As stated in the methods section: typically 1000 shots were summed for MALDI-TOF MS and 5000 for MS/MS. Spectra were processed with the manufacturer’s software (Bruker Flexanalysis 3.3.80) using the SNAP algorithm with a signal/noise threshold of 6 for MS (unsmoothed) and 3 for MS/MS (four-times smoothed).
2. **Search engine, database and fixed modifications:** All glycan data were manually interpreted and no search engine or database was employed; the fixed modification is the 2-aminopyridine label at the reducing end (GlcNAc_1_-PA fragments of *m/z* 300).
3. **Exclusion of known contaminants and threshold:** All glycan data were manually interpreted; only peaks with an MS/MS consistent with a pyridylaminated chitobiose core were included – the ‘threshold’ for inclusion was an interpretable MS/MS spectrum (at least in terms of composition).
4. **Enzyme specificity:** A description of the PNGase Ar release method is given in the methods section; the enzyme should remove N-glycans from glycopeptides regardless of the presence of core α1,3-fucose on the reducing-terminal GlcNAc.
5. **Isobaric/isomeric assignments:** For isomeric species, differences in RP-HPLC elution and MS/MS were used for the assignment (as described in the text).

***Glycan or glycoconjugate identification***

1. **Precursor charge and mass/charge (*m/z*):** All glycans detected were singly-charged. For the positive mode, the *m/z* values are for protonated forms. Depending on the glycan amount or preparation, the relative amounts of the H^+^, Na^+^ and K^+^ adducts varied. Maximally two decimal places used for the *m/z* annotations consistent with the accuracy of MALDI-TOF MS; in the figures and due to space limitations, only one decimal place is presented. Previous data indicate an average +0.03 Da (+ 22 ppm) deviation between the measured and the calculated *m/z* values on the instrument used.
2. **MALDI-TOF MS settings (positive mode):** Ion Source 1 and 2 were 19.00 and 16.75 kV; Lens, 9.00 kV; Reflector 1 and 2, 21.05 and 9.65 kV; Pulsed Ion Extraction, 160 ns; Matrix Suppression typically up to 700 Da; Detector Gain, typically 2163 V.
3. **MALDI-TOF MS/MS settings (positive mode):** Ion Source 1 and 2 were 6.00 and 5.35 kV; Lens, 2.90 kV; Reflector 1 and 2, 27.00 and 11.75 kV; Lift 1 and 2, 19.00 and 4.00 kV; Pulsed Ion Extraction, 140 ns; Detector Gain, typically 2260 V when fragmenting; Laser Power Boost typically 50%; not in CID mode; PCIS typically 0.65%.
4. **All assignments:** For the glycans present in each pool, see the RP-HPLC chromatograms annotated with structures shown according to the Standard Nomenclature for Glycans.
5. **Modifications observed:** Listed are the *m/z* values for glycans carrying a reducing terminal pyridylamine group as judged by presence of an *m/z* 300 GlcNAc_1_-PA fragment. As the glycans are otherwise chemically unmodified, *Δm/z* of 146, 160, 162, 176, 165 and 203 correspond to fucose, methyl-fucose, hexose, methyl-hexose, phosphorylcholine or *N-*acetylhexosamine (positive ion mode). There was no indication for the presence of sulphate, phosphate or sialic acid residues.
6. **Number of assigned masses:** Glycan assignments were not just based on measured mass only, but on the basis of MS/MS corroborated by elution data (*i.e.*, glucose unit of HPLC separation).
7. **Spectra:** Representative annotated spectra (MS and MS/MS) defining structural elements are given in various figures.  In total, MS and/or MS/MS data for approximately 40 defined structures are shown.
8. **Structural assignments:** As noted in the results section, the typical oligomannosidic structures are assigned based on elution time and fragmentation pattern; it is otherwise assumed that the glycans contain a di- or tri-mannosyl core consistent with typical eukaryotic N-glycan biosynthesis and that there is processing by GlcNAc-transferases (GnTI, II and V) and core α1,3/6-fucosyltransferases as in a range of multicellular organisms.

*Synthesis of fluorescein-labelled Man5 (****Man5-Fluo****)*

a) 20.0 mg (42.2 μmol, 1 eq) of 5-carboxyfluorescein-OSu **1** (Sigma-Aldrich) and 16.9 mg (127 μmol, 3 eq) of hexinylamine hydrochloride **2** were dissolved in 844 μL of DMSO and 70.3μL (506 μmol, 12 eq) of triethylamine were added. After 24 h at ambient temperature the mixture was dried in high vaccum. The residue was dissolved in 20 mL of water/acetonitrile 1:4 containing 0.1 % of formic acid. Two portions of 10 mL were purified by solid phase extraction (Waters SepPak C18 Classic, 2×360 mg). Each portion of 10 mL was loaded on to the cartridges follwed by washing with 10 mL of water/acetonitrile 1:4 containing 0.1 % of formic acid. The amide eluted with 10 mL of water/acetonitrile 2:3 containing 0.1 % of formic acid. After lyophilization 11.3 mg of 5-carboxyfluorescin-hexinylamide **3** (24.8 μmol, 58.8 %) were obtained. C_27_H_21_NO_6_ (455.47). LC-MS (RP18, 0-20 % CH_3_CN/H_2_O + 0.1 % formic acid): M_calcd_ = 455.14; M_found_ = 456.04 (M+H)^+^.

b) 1 μL (320 nmol) of TGTA **4** (325 mM in water) and 1 μL (320 nmol) of tetrakis(acetonitrile)copper(I)-hexafluorophosphate (325 mM in DMSO) were mixed under argon and incubated for 30 min. 1.0 mg (794 nmol, 1 eq) of **Man5-N_3_** and 1.08 mg (2.38 μmol, 3 eq) of alkine **3** were dissolved in 8 μL of water under argon. Subsequently, 0.5 μL of the solution containing the preformed Cu-TGTA-complex (80 nmol) were added. After keeping the reaction under argon for 24h the mixture was diluted with 1 mL of water and purified by solid phase extraction (Waters SepPak C18 Classic, 2x360 mg). The solution was loaded on to the cartridges follwed by washing with 10 mL of water. The product was eluted with water/acetonitrile 1:7 containing 0.1 % of formic acid). After lyophilization the procedure was repeated. Yield: 770 μg of **Man5-Fluo** (449 nmol, 56.5 %), C_73_H_98_N_6_O_41_ (1715.59). LC-MS (RP18, 0-20 % CH_3_CN/H_2_O + 0.1 % formic acid): M_calcd_ = 1714.58; M_found_ = 1715.27 [M+H]^+^.

A Scheme of chemical synthesis of fluorescein-labelled Man5 and the 1H-NMR spectrum of compound **3.**


**Supplementary Figure 1.** Alignment of HPLC chromatograms of N2 and tm5400 N-glycans. Aliquots of PA-labelled N-glycans, released by PNGase A, were resolved by a RP-amide reversed phase column on HPLC using dextran oligomers as a standard. N2, dashed line in red; tm5400, solid line in black. MALDI MS spectra of major HPLC fractions (*a*, *b*, *c* and *d*) and tri-/tetra-fucosylated-glycan enriched fractions (*i* to *v*; between 5.6 and 7.0 g.u.) are shown, indicative of the absence of tretrafucosylated structures in tm5400.

**
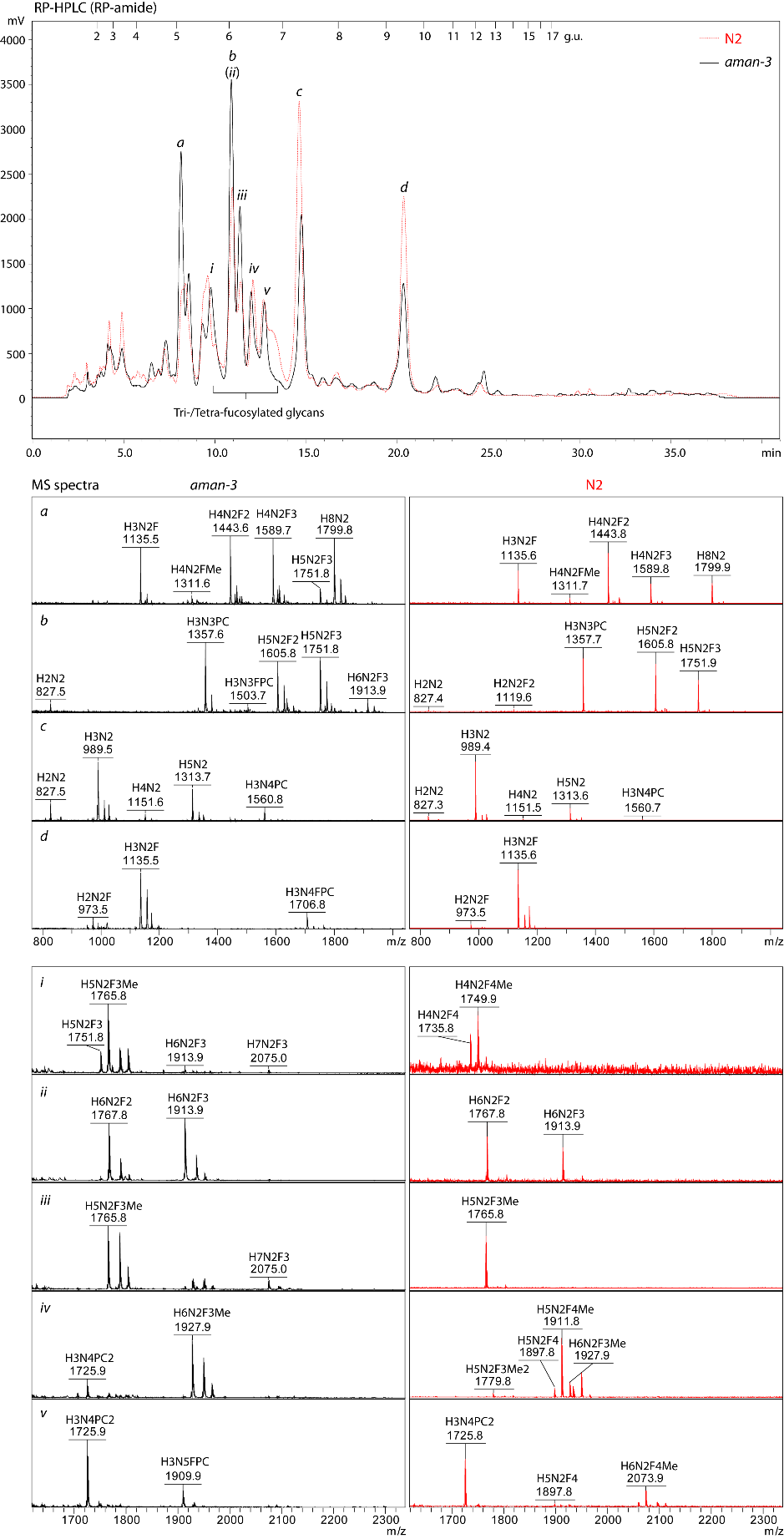
**

**Supplementary Figure 2.** C18 RP-HPLC analysis of a PA-glycan treated with AMAN-3. An aliquot of Man_5_GlcNAc_2_ structure (PA-Man5) purified from a triple GnTI mutant (Trigly) was digested by AMAN-3 and the reactive product displayed a small shift from 17.5 minute to 18.0 minute on the HPLC chromatogram, which co-eluted with a previously characterised structure Man_4_GlcNAc_2_ (PA-Man4) of the Trigly mutant [1]. The loss of one mannose residue from PA-Man5 was confirmed by MALDI-TOF-MS.


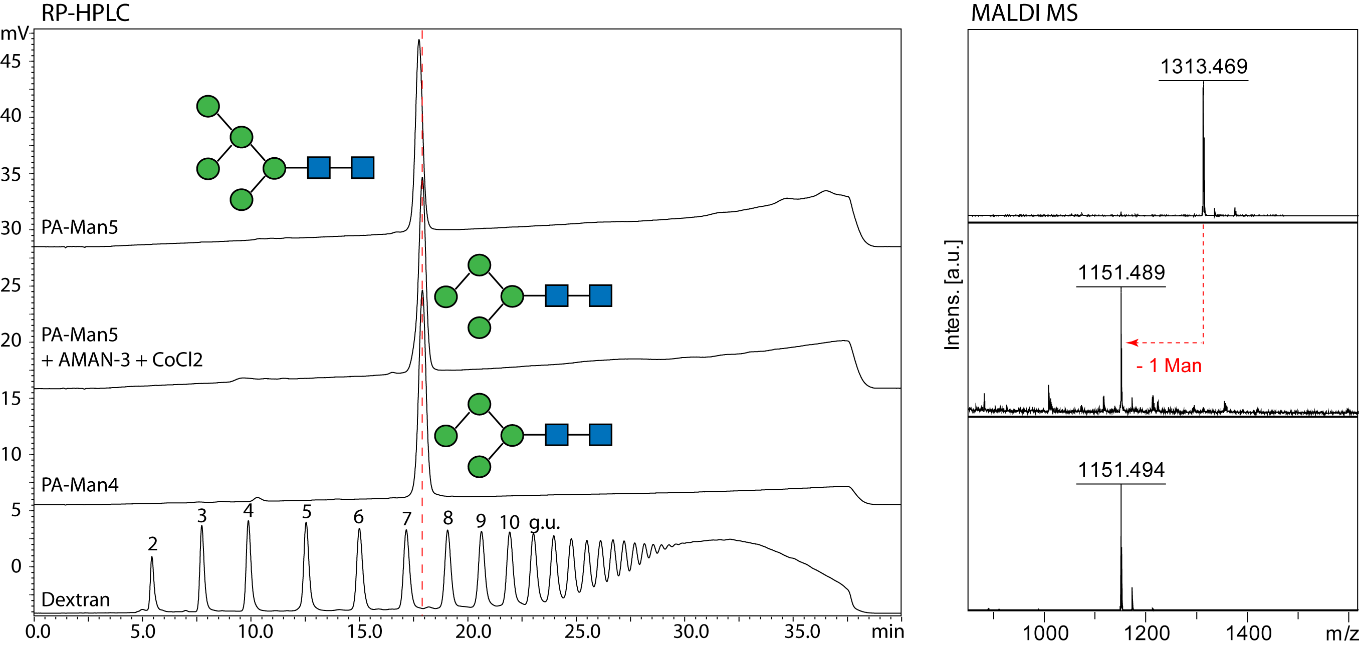


**Supplementary Figure 3.** Co-elution of AEAB-labelled glycans on RP-HPLC. Man_3_GlcNAc_3_ (MGn)was incubated with AMAN-3 in the presence of CoCl2 either at 30°C or at room temperature. Post heat-inactivation, reaction mixtures were analysed on RP-HPLC (**A**) and subsequently, all eluents were examined by MALDI TOF MS/MS (**B** and **C**). Full conversion from Man_3_GlcNAc_3_ to Man_2_GlcNAc_3_ was observed in the sample incubated at 30°C whereas at room temperature, approximately 50% of MGn was digested by AMAN-3 (MS spectra *b-d* in **B**). AEAB-labelled MGn and Man_2_GlcNAc_3_ co-eluted at 21.5 minute.


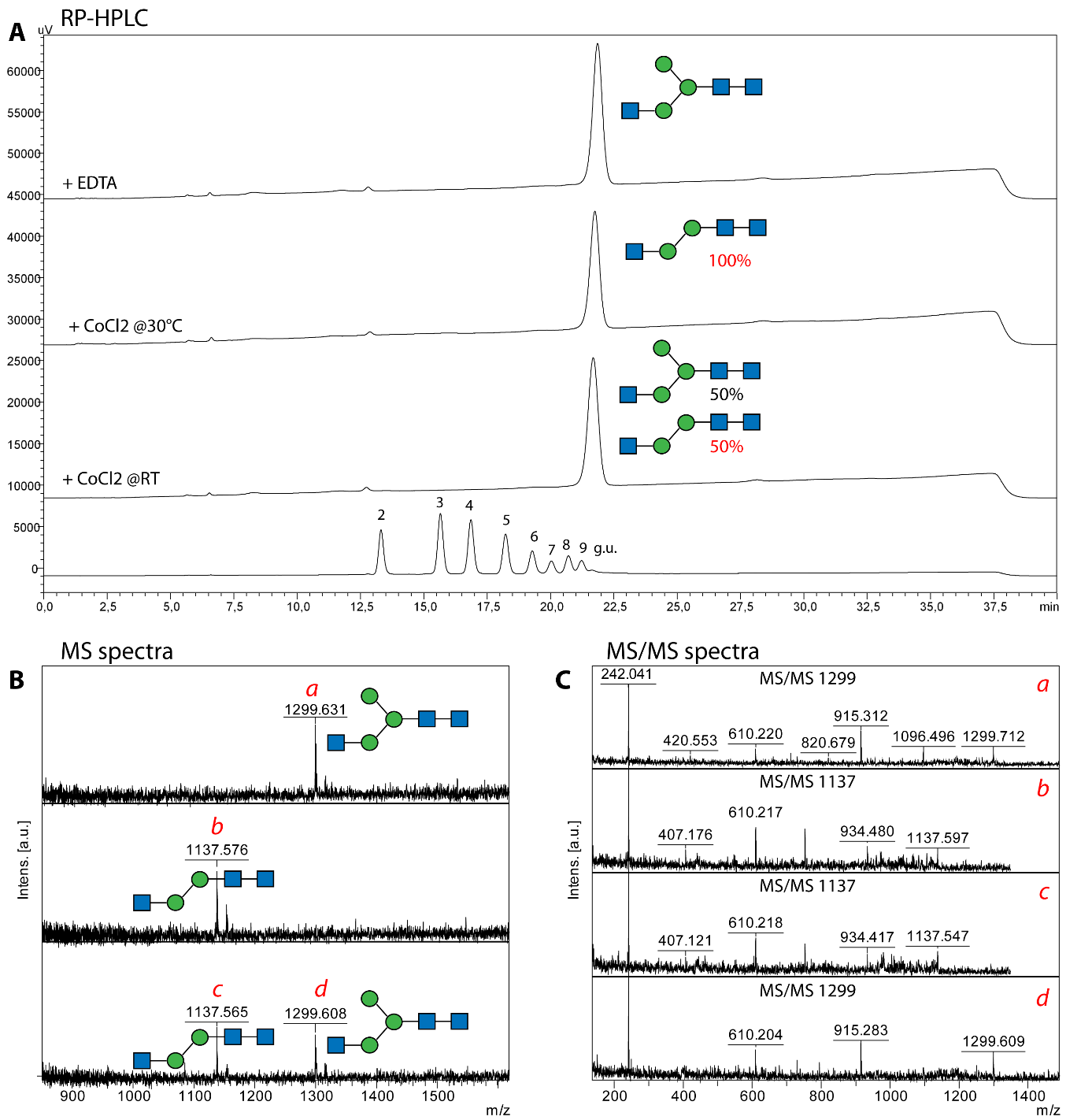


**Supplementary Figure 4.** MALDI-TOF mass spectrometric properties of Man5-Fluo in the positive ion mode. MS spectrum of the intact Man5-Fluo (*m/z* 1737.5, [M+Na]^+^) displayed a series of ions (**A**), possibly due to the “laser-induced degradation”. In comparison to the other MS^2^ spectra of the same compound (**B, D and E**), the fragmentation of the *m/z* 1217.4 ion resulted in the most informative spectrum (**C**, [M+H-linker]^+^), revealing structural details of the glycan portion; whereas fragmentation of the intact sodiated compound (**E**, [M+Na]^+^) resulted in a dramatic loss of the linker (Δ = 498) as well as minor ions indicative of the composition of the glycan. This phenomenon was observed on the other fluorescein-labelled glycans in the remodelling experiment (**Figure 8**), which became the reason for choosing the [M+H-linker]^+^ ions of fluorescein-labelled compounds for MS^2^ fragmentations. N, HexNAc; H, Hexose.

**
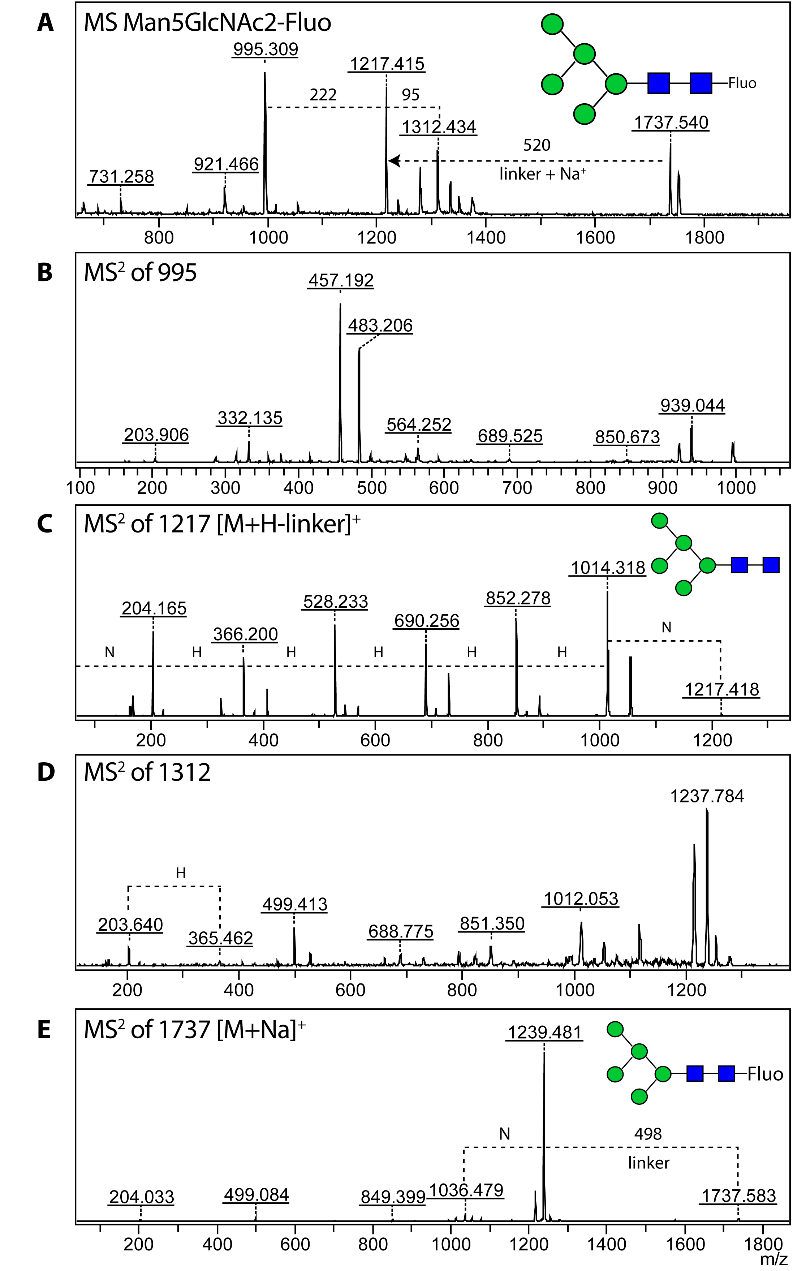
**

**Supplementary Figure 5.** Illustration of body curvature differences between N2 and *aman-3* mutants**.** Snapshots from behavioral movies illustrating animal posture differences between food-deprived N2 and *aman-3* mutants. For each genotype, a single population image from a single assay was randomly selected. The presented illustrative close-up pictures were manually chosen to highlight the difference between a subset of typically N2 postures and specific examples of aman-3 mutants where midbody and tail curvature is obviously reduced.


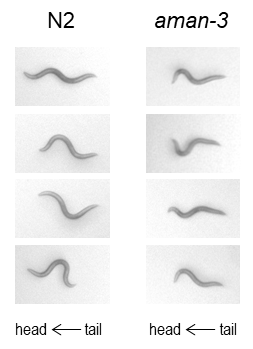


**Supplementary Figure 6.** The gene structure of *C. elegans aman-3.* Deletions in a single mutant (*tm5400*) and triple knockout strains (*hex-2;hex-3;aman-3*, cop1841-cop1844) are marked in red. The CRISPR knock-in region with an insertion of mEGFP-encoding fragment (*egfp* gene being fused to the 19^th^ exon of *aman-3* via a 5×glycine bridge) is marked in blue. CRISPR mutants were verified by and DNA sequencing.


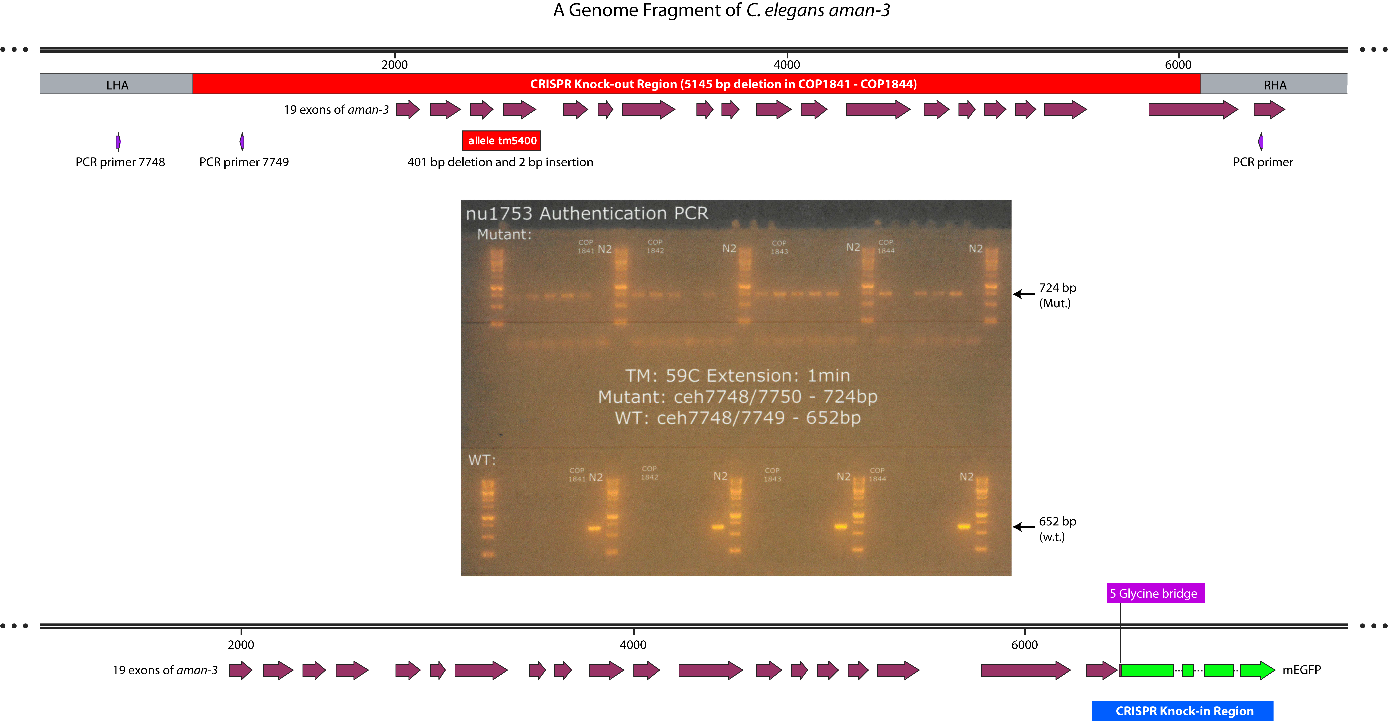


**Supplementary Figure 7.** Plasmid map of the pACEBac1 construct used for the extracellular recombinant production of AMAN-3. The construct was built using Gibson assembly. A synthetic DNA fragment, encoding a N-terminal melittin signal sequence, a HisFLAG tag and a thrombin site, was introduced to the StuI endonuclease restriction site of the original pACEBac1 vector; a truncated *aman-3* DNA fragment was PCR amplified from a pPICZαC construct [2] and ligated in-frame after the thrombin site.


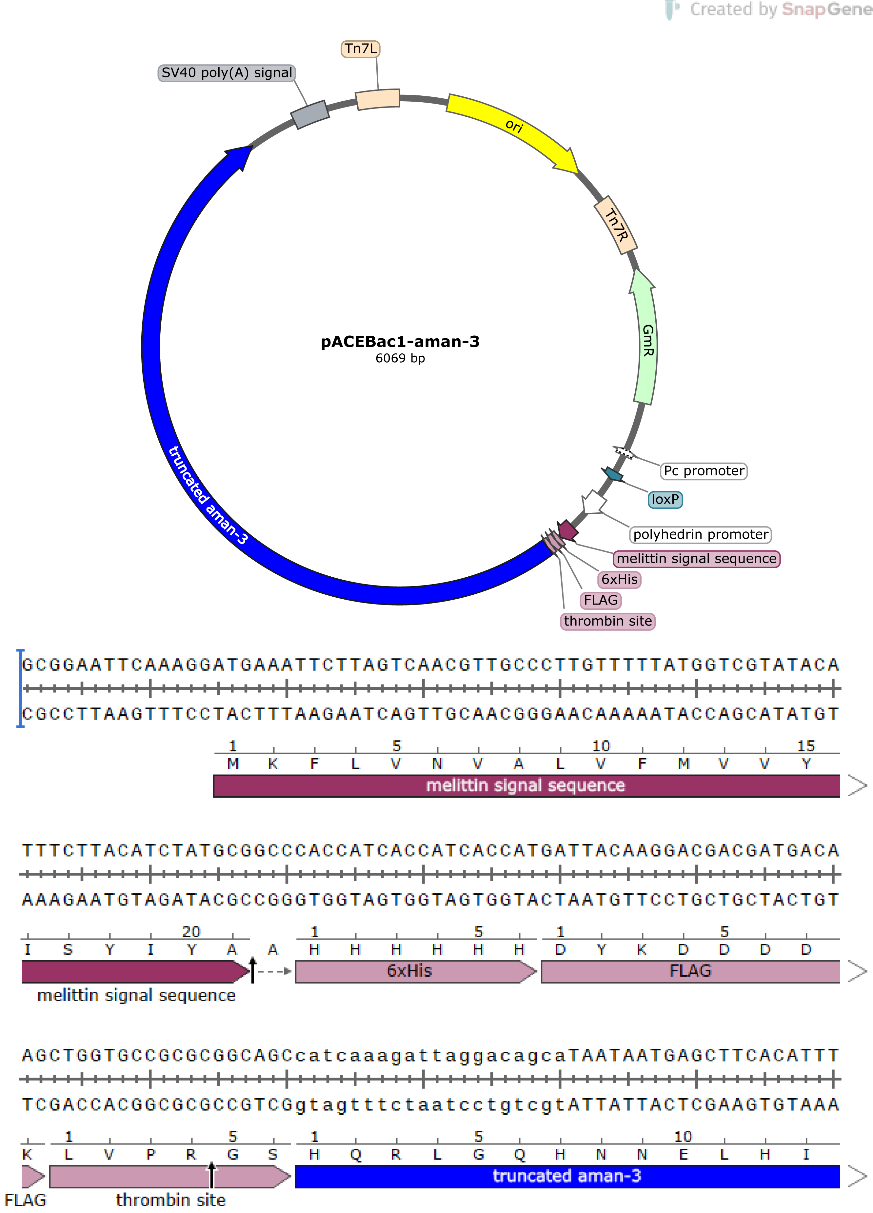


References

1. Yan S, Wang H, Schachter H et al. (2018) Ablation of N-acetylglucosaminyltransferases in Caenorhabditis induces expression of unusual intersected and bisected N-glycans. Biochim Biophys Acta Gen Subj 1862:2191–2203. https://doi.org/10.1016/j.bbagen.2018.07.002

2. Paschinger K, Hackl M, Gutternigg M et al. (2006) A deletion in the golgi alpha-mannosidase II gene of Caenorhabditis elegans results in unexpected non-wild-type N-glycan structures. J Biol Chem 281:28265–28277. https://doi.org/10.1074/jbc.M602878200
